# Placenta Accreta Spectrum: A Narrative Review of Clinical Management, Contemporary Innovations, And Global Challenges

**DOI:** 10.55640/ijmsdh-12-07-04

**Published:** 2026-07-17

**Authors:** Chijioke Ogomegbunam Ezeigwe, George Uchenna Eleje, Uchenna Paschaline Eze, Gerald Tochukwu Igwemadu, Emmanuel Onyebuchi Ugwu, Emmanuel Chukwubuikem Egwuatu, Chigozie Geoffrey Okafor, Chukwuemeka Chukwubuikem Okoro, Chukwudubem Chinagorom Onyejiaka, Obinna Kenneth Nnabuchi, Princeston Chukwuemeka Okam, Adanna Vivian Egwim, Charlotte Blanche Oguejiofor, Malarchy Ekwunife Nwankwo, Chukwunwendu Aloysius Okeke, Chisom God’swill Chigbo, Gerald Okanandu Udigwe, Joseph Ifeanyichukwu Ikechebelu, Akaninyene Eseme Ubom, Kingsley Chidiebere Nwaogu, Ahizechukwu Chigoziem Eke

**Affiliations:** 1 Department of Obstetrics and Gynaecology, Nnamdi Azikiwe University, Awka, Nigeria.; 2 Effective Care Research Unit, Department of Obstetrics and Gynaecology, Nnamdi Azikiwe University, Awka, Nigeria.; 3 Department of Obstetrics and Gynaecology, Nnamdi Azikiwe University Teaching Hospital, Nnewi, Nigeria.; 4 Department of Obstetrics and Gynaecology, Barking, Havering, and Redbridge, University Hospitals NHS, Romford, London, UK.; 5 International Research Center of Excellence, Institute of Human Virology, Nigeria; 6 Department of Obstetrics and Gynaecology, University of Nigeria, Enugu Campus, Nigeria.; 7 Department of Obstetrics and Gynaecology, Faculty of Clinical Sciences, PAMO University of Medical Sciences, Port Harcourt, Nigeria.; 8 Department of Radiology, Lily Hospitals, Warri, Nigeria.; 9 Division of Maternal Fetal Medicine, Department of Gynecology and Obstetrics, Johns Hopkins University School of Medicine, Baltimore, Maryland, USA

## Abstract

**Background:**

Placenta accreta spectrum (PAS) is a severe obstetric complication characterised by abnormal placental adherence or invasion into the uterine wall, posing significant global reproductive health challenges. While clinical guidelines for PAS exist, a critical knowledge gap remains regarding how to successfully translate high-resource diagnostic innovations and multidisciplinary frameworks into resource-limited settings. Furthermore, there is a lack of synthesized literature evaluating how emerging technologies, such as machine learning models and real-time intraoperative flow assessments, can be integrated with traditional imaging to optimise surgical outcomes.

**Objectives:**

This narrative review comprehensively evaluates the evolving clinical landscape of PAS, examining its epidemiology, pathophysiology, risk factors, and diagnostic advancements, while critically appraising multidisciplinary management frameworks across diverse healthcare resource settings.

**Methods:**

A descriptive narrative approach was utilised to synthesise peer-reviewed publications, consensus guidelines, and clinical studies from PubMed, SCOPUS, Web of Science, and Google Scholar. The initial literature search was conducted in March 2026, with an updated search performed in June 2026 during the manuscript revision process. Data synthesis focused on key parameters including study characteristics, diagnostic modalities (ultrasound, magnetic resonance imaging, and emerging imaging mordalities), and clinical management outcomes, with additional context integrated from regional low- and middle-income country (LMIC) reports.

**Results:**

The incidence of PAS has escalated globally, driven primarily by rising primary and recurrent caesarean section rates. Emerging evidence confirms that the condition arises from an endometrial–myometrial interface defect characterised by thinned or absent decidua basalis, accompanied by molecularly driven hypervascularity and altered tissue adhesion. Modern diagnostic signs, such as intracervical lakes (ICL) and the “rail sign,” significantly enhance prenatal identification and predict severe peripartum morbidity. Multidisciplinary care, specialized surgical planning, and intraoperative adjuncts (such as transvaginal ultrasound [TVUS] and machine learning models) improve maternal outcomes, although severe postpartum haemorrhage and maternal mortality remain pronounced in resource-limited systems.

**Conclusion:**

Optimising PAS outcomes requires timely prenatal detection and structured, coordinated care. Advances in ultrasound techniques, combined imaging scores, and real-time intraoperative flow assessments facilitate better risk stratification and surgical optimisation. Addressing current limitations through standardised reporting frameworks and expanding access to subspecialty multidisciplinary care, especially in low-resource settings, remains mandatory to reduce severe maternal morbidity and optimise reproductive health preservation.

## 1.0 Introduction

Placenta accreta spectrum (PAS), historically referred to as morbidly adherent placenta, encompasses a heterogeneous range of pathological conditions characterized by the abnormal adherence or invasion of placental villi into the uterine wall [1,2]. Etymologically derived from the Latin words *ac* and *crescere*, meaning to grow or adhere to, PAS is clinically defined by the failure of the placenta to separate spontaneously from the uterus after childbirth [1,2]. This failure of separation frequently precipitates profuse, torrential haemorrhage, establishing PAS as a primary driver of severe postpartum haemorrhage (PPH), catastrophic maternal morbidity, and peripartum mortality worldwide [1,3, 4]. Indeed, literature indicates that PAS inflicts an 18-fold increase in adverse maternal outcomes at birth, remaining the leading cause of emergency peripartum hysterectomy in Western countries [3, 4].

The underlying pathophysiology of PAS centers on a fundamental disruption of the maternal-fetal interface. Rather than being driven by intrinsically hyper-aggressive trophoblastic behaviour, the condition is primarily an iatrogenic consequence of a compromised uterine wall [1]. The primary structural catalyst is a structural or functional defect in the endometrial–myometrial interface, resulting in defective decidualisation and the total or partial absence of both the decidua basalis and the normal Nitabuch’s proteolytically protective layer [1]. Any process that disrupts or scars the endometrium elevates the risk of this decidual failure, with caesarean birth serving as the most common and pervasive cause [1]. In the absence of this protective decidual buffer, the anchoring chorionic villi bypass normal biological boundaries, attaching directly to or embedding deeply within the scarred, highly vascular myometrium [1]. While traditional classification systems based strictly on the depth of villous invasion are increasingly viewed as outdated, PAS continues to be categorised into three progressive phenotypes based on anatomical penetration [2]: placenta accreta vera: chorionic villi attach directly to the superficial myometrium without intervening decidua; placenta increta: chorionic villi deeply invade the myometrial muscle fibers; and placenta percreta: chorionic villi penetrate entirely through the uterine serosa and may invade adjacent pelvic organs, most commonly the urinary bladder [2].

To mitigate the substantial surgical risks and life-threatening haemorrhages associated with manual placental removal attempts at birth, accurate prenatal diagnosis and calculation of pretest probability are absolutely critical [4,5]. Antenatal imaging serves as an essential screening tool to ensure high-risk women are appropriately triaged to specialised tertiary centers equipped with expert multidisciplinary teams [5]. Current international guidelines recommend offering targeted, specialist placental ultrasound assessments to asymptomatic women with risk factors between 24 and 28 weeks of gestation [1, 5]. While gray-scale and colour Doppler ultrasound remains the first-line diagnostic modality, magnetic resonance imaging (MRI) possesses highly complementary diagnostic value. However, given its high operational cost and reliance on specialised subspecialty interpretation, MRI should primarily be utilised as an imaging adjunct following an initial specialist ultrasound assessment [5]. Modern clinical management is thus increasingly focused on decoding the microvascular biology of the utero-placental interface to improve prenatal risk stratification and optimise intraoperative surgical strategies [3].

Despite these paradigm shifts in understanding and diagnosing PAS, significant gaps persist in the collective clinical literature. Existing reviews predominantly detail management frameworks tailored to well-funded, tertiary-care academic centers in high-income countries. Consequently, there is a stark deficit in synthesised guidance on how to operationalise these advanced protocols in low- and middle-income countries (LMICs), where blood banking systems, interventional radiology, and subspecialty multidisciplinary teams are severely limited or entirely absent. Furthermore, while rapid technological innovations, ranging from novel sonographic markers like “intracervical lakes” to machine learning-based risk-scoring models, have recently emerged, their comparative efficacy and clinical implementation boundaries have not been systematically synthesised. This narrative review comprehensively evaluates the evolving clinical landscape of PAS, examining its epidemiology, pathophysiology, risk factors, and diagnostic advancements, while critically appraising multidisciplinary management frameworks across diverse healthcare resource settings.

## 2.0 Methods

### 2.1 Study design and search strategy

This narrative review was conducted in accordance with the Scale for the Assessment of Narrative Review Articles (SANRA) recommendations to ensure methodological transparency and scientific rigor [6]. The approach was adopted to synthesize current literature regarding the epidemiology, pathogenesis, diagnosis, and management of PAS. A comprehensive literature search was conducted across the PubMed, SCOPUS, web of Science, and Google Scholar databases for peer-reviewed articles, international consensus statements, and clinical guidelines. The initial literature search was conducted in March 2026, with an updated search performed in June 2026 during the manuscript revision process. The electronic search strategy utilised combinations of MeSH terms and keywords, including “placenta accreta spectrum,” “morbidly adherent placenta,” “diagnosis,” “management,” and “prevention.” To ensure global contextual relevance and address resource-specific clinical outcomes, the search was supplemented with regional data and institutional reports, with a particular focus on sub-Saharan Africa. Furthermore, the reference lists of all retrieved articles, reviews, and societal guidelines were manually screened to identify additional relevant publications.

### 2.2 Study selection and data synthesis

Literature eligibility was restricted to English-language publications that provided substantive insights into the understanding, diagnostic optimisation, or therapeutic protocols of PAS. Studies were selected based on their methodological relevance and overall contribution to the evolving landscape of the disease. Conversely, articles focusing on unrelated participant populations, extraneous obstetric interventions, or those lacking clear clinical endpoints were excluded.

The literature search initially identified 339 citations. After removing duplicate records and screening the remainder for relevance, 135 full-text articles were reviewed. Ultimately, 90 publications met the eligibility criteria and were included in this narrative review. The key data points from the selected literature, encompassing study designs, women cohorts, diagnostic interventions, and primary clinical findings, were extracted and summarised descriptively within a structured framework (Table 1). In accordance with standard narrative review methodologies, no formal critical appraisal of study quality or quantitative meta-analysis was performed.

## 3.0 Epidemiology and incidence of PAS

The prevalence of PAS has increased dramatically over the past five decades, largely in parallel with rising global caesarean section rates [5]. This escalating rate of caesarean births directly drives the incidence of PAS, as recurrent uterine surgeries induce endometrial damage and disrupt vascular architecture, predisposing women to abnormal placental attachment [1, 7]. Chronologically, the reported rate of PAS was 1 in 4,000 births in the 1970s and 1 in 2,500 births in the 1980s [8]. Contemporary data from the United States demonstrate a stark rise, revealing an incidence ranging between 1 in 533 [9] and 1 in 730 births [10], which represents an approximate eightfold increase since the 1970s [1, 7].

PAS occurs in approximately 0.17% of pregnancies within the general obstetric population, though sub-populations face significantly higher risks. For instance, a study conducted in South Eastern Nigeria identified a local incidence of 1 in 282 births (0.35%) [11]. Women with a history of prior caesarean birth experience a sevenfold higher risk of developing subsequent PAS compared to the unscarred population [1–3]. This risk increases scale-dependently with the number of prior caesarean births: the probability rises from approximately 1–5% in cases of isolated placenta previa without previous surgery to as high as 60–67% when placenta previa coexists with three or more previous caesarean births [12]. Significant regional variations exist within the literature. Higher prevalence rates are typically documented in high-income countries due to superior diagnostic capacity and robust institutional reporting systems [1,2]. Conversely, in low- and middle-income countries (LMICs), including sub-Saharan Africa, the true burden of disease is systematically underestimated due to limited prenatal diagnostic infrastructure and poor documentation [13].

### 3.1 Interpregnancy interval and incidence of PAS

To assess the specific impact of the interpregnancy interval (IPI) on the incidence of PAS, Umeh et al. conducted a prospective, three-centre cohort study involving parturients with at least one prior caesarean section who reached a gestational age of 34 weeks or more [14]. The study recorded a PAS incidence of 1.6% in the short IPI cohort compared to 0.8% in the normal IPI cohort (odds ratio [OR] = 2.02; 95% confidence interval [CI] = 0.18–22.13; p = 0.57) [14]. The authors concluded that a short interpregnancy interval does not significantly alter the incidence of PAS following a caesarean birth. However, they noted that further large-scale, well-powered studies are required to corroborate these findings within low- and middle-income clinical settings [14].

## 4. Classification and diagnostic criteria

### 4.1 International Federation of Gynaecology and Obstetrics (FIGO) Classification

In 2019, FIGO introduced a formal clinical and histological classification system to refine diagnostic consistency, stratifying PAS into three distinct severity grades [15]: Grade 1: abnormally adherent placenta (placenta accreta vera); Grade 2: abnormally invasive placenta (placenta increta) and Grade 3: abnormally invasive placenta (placenta percreta).

To standardise research outcomes, the women are stratified into three comparative cohorts based on these criteria [15]: Group A: cases meeting clinical criteria only (Grades 1–3); Group B: cases meeting histological criteria (Grades 1–3), defined microscopically by a total lack of intervening decidua between the villous tissue and the uterine myometrium, with or without corresponding clinical signs. Cases meeting both clinical and histological definitions are preferentially assigned here and Group C: control cases demonstrating neither clinical nor histological evidence of PAS [15].

### 4.2 Topographic invasion mapping at caesarean section

To refine intraoperative staging, objective topographic invasion areas observed during caesarean section are classified into four distinct anatomical types [16]: **Type 1:** The placenta reaches or extends beyond the uterine serosa, demonstrating prominent neovascularisation in direct relation to the upper posterior wall of the urinary bladder; **Type 2:** the placenta invades the lower posterior bladder wall, demonstrating extensive trigone-cervical invasion and pathological collateral blood supply bridging the bladder, cervix, and vagina; **Type 3:** the placenta reaches or extends beyond the uterine serosa, accompanied by lateral parametrial placental invasion and **Type 4:** the placenta reaches or extends beyond the posterior serosa localised strictly at the lower uterine segment.

## 5. Pathophysiology and aetiopathogenesis

The development of PAS hinges on a fundamental structural and molecular disruption of the maternal-fetal interface. Rather than being driven by intrinsically malignant or hyper-aggressive trophoblastic behaviour, PAS is primarily a secondary consequence of a mechanically or functionally compromised uterine wall [17].

### 5.1 The Endometrial–myometrial interface defect

In a physiologically normal pregnancy, the endometrium undergoes a highly regulated transformation into the decidua basalis. This specialised layer serves as a protective, immunologically active buffer that limits the depth of trophoblastic penetration [18]. The primary structural catalyst for PAS is a localized structural or functional defect at the endometrial–myometrial interface, culminating in a failure of normal decidualisation. Consequently, this protective decidual buffer is either severely thinned, functionally incompetent, or entirely absent [18]. This decidual failure occurs most frequently at sites of previous uterine trauma where prior surgeries have permanently disrupted the local vascular architecture and tissue layer integrity. Pervasive clinical triggers include prior caesarean births (the single highest independent risk factor), rigorous uterine curettage, myomectomy, and a history of manual placental removal [18].

### 5.2 Disruption of normal placentation

Healthy placentation relies on a tightly synchronised physiological cascade involving regulated trophoblast invasion, the extensive remodelling of maternal spiral arteries to optimise low-resistance blood flow, and precise local immune modulation to prevent maternal rejection of the allograft [19]. When the decidua is damaged or absent, this regulatory feedback loop is broken. Normal boundary controls disappear, allowing anchoring chorionic villi to bypass the endometrial boundary entirely. At the microscopic level, placental villi attach directly to, or deeply embed within, the underlying myometrial muscle fibres instead of interacting with decidual cells. Because myometrial tissue completely lacks the natural cleavage plane provided by the decidua, the placenta becomes structurally locked into the uterine wall, completely preventing normal physiological separation after childbirth [19].

### 5.3 Molecular mechanisms of hyperinvasiveness

While the initial trigger is architectural damage (the missing decidual buffer), secondary local molecular alterations quickly follow. These alterations create a microenvironment that actively promotes deep tissue invasion and massive collateral blood vessel growth [20, 21]: The pathophysiology of PAS is primarily related to a defect in the endometrial–myometrial interface, resulting in failure of normal decidualization, particularly at sites of uterine scarring. This defect permits abnormal trophoblastic invasion into the myometrium [17]. Normal placentation involves tightly regulated trophoblast invasion, spiral artery remodeling, and immune modulation; disruption of these processes leads to abnormal placental adherence.

At the microscopic level, placental villi anchor directly to myometrial fibers rather than decidual cells, preventing normal placental separation after delivery. Molecular mechanisms include dysregulated angiogenesis, altered expression of adhesion molecules such as E-cadherin, and increased vascular endothelial growth factor activity, contributing to hyperinvasiveness of trophoblasts [Table 2].

As established by Shteynman et al., this combined breakdown of normal regulatory boundaries, characterised by a missing decidual layer, dysregulated blood vessel growth, and altered cellular adhesion, transforms a localized structural scar into a hyperinvasive, highly vascularised, and life-threatening clinical condition [17].

## 6.0 Risk factors, clinical presentation, and diagnostic advancements

### 6.1 Risk factors for PAS

The primary aetiological drivers of PAS are intimately linked to prior mechanical or functional uterine trauma. Surgical risk factors encompass prior caesarean sections, myomectomies, endometrial ablation, and recurrent uterine dilation and curettage. Cases have also been documented following conservative, fertility-sparing surgical interventions for ovarian malignancies [22]. Non-surgical predisposing factors include advanced maternal age, multiparity, congenital uterine anomalies, assisted reproductive technologies (ART), maternal smoking, and pelvic irradiation [22]. In a landmark prospective cohort study comprising 4,146 pregnant women, Imafuku et al. identified a local PAS incidence of 2.1% (n = 87) [23]. Multivariable logistic regression analysis demonstrated that both surgical and non-surgical variables independently correlate with the development of PAS, revealing that a prior history of caesarean birth carries an odds ratio of 3.3, a previous dilation and curettage carries an odds ratio of 2.8, prior hysteroscopic surgery carries an odds ratio of 5.7, and previous uterine artery embolisation carries an exceptionally high odds ratio of 44.1 [23]. Furthermore, the same multivariable analysis indicated that conception via assisted reproductive technology carries an odds ratio of 4.1, while the concomitant presence of placenta previa in the current pregnancy carries an odds ratio of 13.1 [23]. The authors concluded that the presence of these overlapping clinical markers shifts women into a high-risk category requiring targeted antenatal screening registries [23].

### 6.2 Clinical presentation of PAS

PAS is characteristically asymptomatic during the antenatal period and is typically uncovered via routine screening or specialised third-trimester imaging. Consequently, a substantial proportion of women present unexpectedly during the intrapartum phase, which is defined by failure of placental separation, retained placental fragments, and sudden, life-threatening PPH upon manual removal attempts [24]. Secondary clinical signals include fetal malpresentation and structural complications secondary to a low-lying placenta or placenta previa [24]. In resource-replete Western countries, PAS stands as the leading cause of emergency peripartum hysterectomy [24]. The clinical phenotype of placenta percreta represents a significantly higher risk of severe maternal morbidity relative to superficial accreta or increta subtypes [7]. Thus, accurate prenatal mapping of the topological depth of invasion is mandatory to facilitate referral to centralised tertiary hubs, reducing intraoperative surgical complications through coordinated multidisciplinary care [24].

### 6.3 Diagnostic modalities: ultrasound and magnetic resonance imaging for PAS

Despite diagnostic advancements, up to 50% of PAS cases escape prenatal detection globally. Cultivating a high index of clinical suspicion during routine obstetric scans is vital to initiate pre-surgical optimisation. Ultrasound remains the primary diagnostic tool; transvaginal ultrasound offers superior resolution and sensitivity over transabdominal ultrasound, particularly for verifying lower uterine segment involvement. Characteristic 2D sonographic hallmarks include the loss or irregularity of the retroplacental hypoechoic zone, multiple tortuous intraplacental lacunae imparting a classic Swiss cheese architectural appearance to the placental parenchyma, marked thinning of the retroplacental myometrium to less than 1 mm, and interruption or hypervascular hyperlucency at the uterine serosa–bladder interface [24,25]. Colour Doppler imaging enhances diagnostic precision by demonstrating turbulent lacunar blood flow, abnormal bridging vessels traversing the outer myometrium, and extreme hypervascularity at the uteroplacental boundary [26].

MRI serves as a diagnostic adjunct reserved for cases with equivocal ultrasound findings, isolated posterior placentation, or suspected deep parametrial and pelvic organ invasion. Signature T2-weighted MRI markers include structural uterine bulging, heterogeneous placental signal intensity, and dark intraplacental bands [26]. However, the diagnostic sensitivity of these modalities varies considerably across the literature, ranging from 31% to 91% for ultrasound and 54% to 97% for MRI [26]. The American College of Obstetricians and Gynaecologists (ACOG) highlights that it remains unclear whether routine MRI integration improves maternal outcomes over specialist ultrasound mapping alone [26,27]. To resolve these diagnostic inconsistencies, Pain et al. designed and validated a multi-modal nomogram designated as the Percreta Score, which combines specific ultrasound and MRI indices to significantly enhance the positive predictive value when differentiating focal accreta from invasive placenta percreta [28]. In contrast, Einerson et al. noted that isolated MRI interpretations could occasionally yield misleading over-diagnoses [29], whereas Romeo et al. maintained that ultrasound and MRI function as complementary imaging tools to accurately gauge the biological boundary of invasion [30]. Imaging accuracy depends heavily on operator experience. Khwankaew et al. evaluated the efficacy of combining TAS with adjunctive TVS among operators with disparate experience levels and noted that combining TAS and TVS yielded excellent interobserver reliability with a kappa value of 0.92 [31]. This approach significantly increased the diagnostic accuracy of the lower-experienced operator from 81.40% to 94.30%, and that of the expert operator from 94.30% to 97.10%, validating the clinical utility of multi-modal ultrasound frameworks in standardising diagnostic performance [31].

### 6.4 Emerging diagnostic vectors: machine learning and real-time evaluation of PAS

Recent investigative pipelines have introduced advanced technological adjuncts to enhance antenatal and intraoperative diagnostic precision. Jochum et al. leveraged machine learning (ML) models to synthesize longitudinal, trimester-specific haematological trends, historical clinical indicators, and sonographic signs across 186 PAS cases and 217 high-risk controls [33]. Multivariable logistic regression identified prior caesarean birth with an odds ratio of 1.8, and second- or third-trimester ultrasound markers with odds ratios of 28.1 and 27.6, respectively, as robust disease predictors. Interestingly, third-trimester mean platelet volume (MPV) was inversely associated with PAS presence, demonstrating an odds ratio of 0.55 [33]. The resulting machine learning algorithms achieved high diagnostic performance, where Model 1 predicted definitive PAS with 90.0% accuracy, Model 2 utilised early gestational haematological shifts to achieve 88.8% accuracy, and Model 3 predicted severe peripartum haemorrhage exceeding 1,500 mL with an accuracy of 74.3% [33]. These findings suggest that integrating computational ML models can significantly improve pre-surgical risk stratification and institutional resource allocation [33].

Intraoperatively, real-time diagnostic indicators are equally vital for defining the safety margin before extirpative surgery is initiated. Mitoma et al. evaluated the utility of intraoperative TVUS tracking the blood flow persistence time within cervical hypervascular zones following fetal delivery [32]. In a cohort where 38.5% of cases had confirmed PAS, the post-birth duration of low-velocity colour Doppler signals was significantly longer in PAS cases compared to non-PAS controls [32]. Receiver operating characteristic curve analysis demonstrated exceptional discrimination with an area under the curve between 0.94 and 0.95, indicating that a flow persistence threshold of less than 7 minutes ruled out PAS with 100% sensitivity, whereas a threshold of 14 minutes or more yielded a 100% positive predictive value for PAS and 96% specificity for predicting the necessity of a peripartum hysterectomy [32]. This real-time sonographic parameter serves as a reliable intraoperative indicator to guide surgical choices between immediate uterine preservation or planned extirpation [32].

### 6.5 Definitive staging and advanced sign descriptors of PAS

A definitive diagnosis of PAS is confirmed postnatally via clinical or histopathological endpoints. For patients undergoing planned or emergency hysterectomy, histopathology serves as the gold standard, requiring visual verification of chorionic villi directly invading the myometrium without intervening decidua [24]. For patients managed via conservative, non-extirpative approaches, a clinical diagnosis is established when no cleavage plane can be identified between the placenta and the lower uterine segment during active management of the third stage of labour [24]. To further refine sonographic staging, modern investigations have characterized two high-resolution ultrasound signs known as the rail sign and the tramline sign [34,35]. While phonetically similar, these terms describe distinct aspects of advanced placental invasion [36,37].

The rail sign, formulated by Shih et al., is a 2D colour Doppler finding that denotes extensive subplacental and uterovesical hypervascularity and manifests as two parallel hypervascular lines flanking the uterovesical space, with perpendicular bridging collaterals mimicking railway sleepers [36]. The presence of a positive rail sign denotes deep villous invasion and carries a significantly higher risk of a true invasive placenta increta or percreta compared to rail-negative cohorts, affecting 83.3% versus 27.9% of patients respectively [38]. In contrast, the tramline sign, formulated by Dall’Asta et al., is derived from offline 3D ultrasound volume analysis utilising contrast-enhancing rendering algorithms without colour Doppler [38]. By bisecting the myometrium and bladder perpendicularly in the sagittal plane, it delineates two structural boundaries where an inner tramline represents the myometrial–placental interface and an outer tramline represents the uterine serosa–bladder interface [38]. Disruption of the proximal internal tramline indicates superficial invasion such as placenta accreta or increta, whereas concurrent disruption of the distal external tramline confirms structural serosal breakdown and bladder mucosal involvement typical of placenta percreta [38]. A partially or fully obliterated tramline sign exhibits 100% sensitivity for PAS detection and correlates strongly with markers of extreme intraoperative complexity and the inevitability of peripartum hysterectomy [34,38].

## 7.0 Management strategies for PAS

### 7.1 Clinical principles and delivery planning for PAS

The clinical optimisation of PAS dictates early prenatal identification and the strict implementation of a coordinated, multidisciplinary approach. Comprehensive antenatal care involves detailed patient counseling, frequent sonographic or clinical monitoring, and timely referral to specialised tertiary centers possessing dedicated expertise in complex pelvic surgery and massive transfusion protocols. Surgical planning standardly mandates arranging a scheduled caesarean birth between 34 0/7 and 35 6/7 weeks of gestation, a window optimised to precede the onset of spontaneous labour or acute haemorrhage while ensuring fetal maturity [39].

### 7.2 Definitive surgical management: planned caesarean hysterectomy for PAS

The gold standard definitive treatment for extensive or invasive PAS remains a planned peripartum caesarean hysterectomy with the placenta left completely in situ. Attempting manual separation of an abnormally adherent placenta invariably disrupts hypervascular collateral beds, instigating catastrophic hemorrhage. The surgical protocol requires a wide midline vertical skin incision or a generous transverse approach to optimise visualization of the lower uterine segment and retroperitoneal spaces. The uterine incision must be strategically placed fundally or high on the anterior corpus, completely clear of the upper placental border, confirmed via preoperative or intraoperative ultrasound. Following fetal extraction, the umbilical cord is ligated and cut close to its insertion, and the uterine incision is rapidly closed with the placenta contained securely inside the uterine cavity [16, 17, 39].

Dissection proceeds with meticulous retroperitoneal mobilisation. Early development of the paravesical and pararectal spaces is imperative to map altered pelvic anatomy and isolate aberrant collateral vessels. Uterine devascularization is systematically achieved through retroperitoneal ligation of the anterior divisions of the internal iliac arteries or selective uterine artery ligation. When extensive bladder invasion (placenta percreta) is encountered, a deliberate intentional cystotomy may be executed, or a total/subtotal hysterectomy performed while intentionally leaving a focal plaque of adherent placental tissue attached to the posterior bladder wall to prevent extensive lower urinary tract disruption [39].

### 7.3 Conservative and uterus-preserving management approaches for PAS

In highly selected women who express a strong desire for future fertility, and where focal or superficial involvement permits, conservative, uterine-preserving approaches may be pursued. The primary rationale behind conservative management is the complete avoidance of peripartum hysterectomy and its attendant psycho-emotional and reproductive impacts [40]. Under this paradigm, the placenta is intentionally left entirely in situ after child birth, relying on subsequent autolytic degradation over several months, often accelerated by adjunctive uterine artery embolisation (UAE) or selective localized resection. However, this approach carries a substantial risk profile, demanding stringent post-operative surveillance due to documented complications including delayed catastrophic haemorrhage, severe pelvic sepsis, disseminated intravascular coagulation, and the high eventual probability of secondary emergency hysterectomy [40].

#### 7.3.1 Expectant management and adjunctive methotrexate therapy for PAS

Expectant management involves leaving the entire placenta undisturbed in situ after fetal delivery, closing the hysterotomy incision, and completely eschewing any manual separation attempts [41]. This non-extirpative approach relies on gradual, spontaneous autolytic biodegradation and placental resorption over weeks to months, requiring intensive long-term postpartum surveillance. While overall success rates of up to 78% have been documented, this path carries profound secondary risks, including delayed catastrophic haemorrhage, systemic sepsis, uterine necrosis, and the eventual requirement for emergency delayed hysterectomy. Women must be monitored through structured longitudinal protocols incorporating serial colour Doppler ultrasound or magnetic resonance imaging alongside quantitative serum beta-human chorionic gonadotropin (beta-hCG) tracking to confirm functional placental involution [41].

Historically, systemic or localized methotrexate therapy has been utilized as an adjunct to expectant management to accelerate placental resorption by targeting and disrupting proliferating trophoblastic tissue [42]. However, empirical evidence supporting its clinical efficacy remains limited and highly inconsistent. While isolated small case series suggest a shorter time to complete placental resorption and a reduced necessity for secondary hysteroscopic or surgical procedures, comparative trials report no significant statistical benefit over expectant management alone [42]. Furthermore, the clinical utility of methotrexate in the postpartum phase is complicated by severe dose-dependent maternal risks, including hepatotoxicity, profound myelosuppression, and debilitating mucositis. Consequently, contemporary consensus guidelines do not recommend routine prophylactic methotrexate administration in PAS, advising that it only be considered selectively on an individualised basis where compliant, close follow-up is guaranteed [42].

#### 7.3.2 Localised segmental resection for PAS

Segmental uterine resection, also characterised as one-step conservative surgery, involves the complete localised excision of the invaded myometrial block along with its adherent placenta, followed by immediate, multi-layered reconstructive repair of the uterine wall. This technique is most appropriate for focal, well-demarcated PAS lesions possessing clear anatomical margins of invasion, and it demands an experienced surgical team capable of handling complex pelvic reconstruction. Compared to primary caesarean hysterectomy, this approach successfully preserves fertility but exposes the woman to substantial risks of primary intraoperative haemorrhage, localised uterine scar rupture or dehiscence in subsequent pregnancies, and the need for immediate reoperation if the initial borders of invasion are misjudged [43]. Clinicians face three clear treatment paradigms for PAS: caesarean hysterectomy, expectant management, and uterine-sparing surgical techniques. One-step conservative surgery represents the most extensively described uterus-preserving surgical option in the literature, possessing strong empirical evidence validating its safety; however, relatively few surgical groups apply it globally, largely due to a widespread clinical misconception that it represents an overly complex procedure requiring inaccessible training pipelines applicable to only a minority of patients [43].

#### 7.3.3 The Triple-P procedure

The Triple-P strategy is a highly structured, three-step conservative surgical approach formulated in 2010 as a novel alternative to peripartum hysterectomy to avoid severe maternal morbidity and mortality [44,45]. The protocol comprises three sequential steps: perioperative placental localisation: ultrasound mapping of the superior placental edge, followed by a uterine incision made strictly above this upper border; pelvic devascularisation: preoperative placement of internal iliac or uterine artery occlusion balloons to control pelvic blood flow; and placental non-separation and myometrial excision: Direct en bloc excision of the entire unseparated, invaded myometrial segment, followed by structural uterine reconstruction. Multiple modifications to the original Triple-P procedure have been developed globally to achieve effective pelvic devascularisation based on locally available institutional resources [45].

Mappa et al. conducted a comprehensive systematic review to determine the comparative effectiveness of the classic Triple-P procedure versus its various technical modifications in reducing estimated blood loss (EBL) and the rate of peripartum hysterectomy in women diagnosed with PAS [46]. The authors performed a structured literature search across PubMed, Embase, and Google Scholar using the indexing terms “Triple P” and “Modified Triple P,” with retrieved papers independently assessed for data extraction and content analysis. The analytical framework tracked total case volumes, total EBL, blood transfusion rates, accidental injury to adjacent pelvic organs (urinary bladder, ureter, or bowel), the necessity for secondary embolisation, ICU admissions, postoperative length of hospital stay, the rate of secondary peripartum hysterectomy, and the precise nature of any surgical modifications.

The search identified six eligible articles evaluating the classic Triple-P procedure and 8 articles evaluating the modified Triple-P procedure [46]. Within the classic cohort, 75 patients underwent the Triple-P procedure, demonstrating an estimated mean blood loss of 2.31 L, a blood transfusion rate of 52%, a low accidental bladder injury rate of 1.3%, and a 0% rate of secondary peripartum hysterectomy. Conversely, within the modified cohort, 654 women underwent a modified Triple-P protocol, demonstrating an estimated mean blood loss of 1.4 L, a blood transfusion rate of 64.5%, a mean postoperative inpatient stay of 3.86 days, and a secondary peripartum hysterectomy rate of 6.1%. The authors concluded that both the classic Triple-P and modified Triple-P procedures are associated with significantly lower estimated blood loss compared to historical rates documented for primary caesarean hysterectomy. The classic Triple-P procedure demonstrated lower rates of accidental injury to the urinary bladder and ureters compared to the modified protocols and traditional hysterectomy data, while both approaches maintained low rates of secondary peripartum hysterectomy (0% and 6.1%, respectively) [46].

#### 7.3.4 Uterus-preserving focal excision

Vural et al. evaluated the feasibility, clinical safety, and long-term outcomes of a modified uterus-preserving focal excision technique designed for high-grade PAS, including placenta percreta, which operates based on defining local pathoanatomical defects and collateral neovascularisation rather than macroscopic tissue invasiveness [47]. The authors retrospectively reviewed 15 consecutive cases of high-grade PAS (FIGO Stages 2 to 3b) treated between 2020 and 2025 at a tertiary referral centre using a standardised focal resection protocol. Antenatal diagnosis was established using transabdominal and transvaginal ultrasonography, and preoperative planning required detailed counselling of the women, multidisciplinary team coordination, and advanced preparation of blood products. The surgical technique involved meticulous retroperitoneal bladder dissection, stepwise ligation of pelvic collateral vessels, and targeted excision of the focal placenta percreta zones.

Uterus-preserving surgery was successfully achieved in 14 of the 15 women (93%), while one woman (7%) required an emergency supracervical hysterectomy due to unmanageable intraoperative haemorrhage [47]. The median blood loss was 2000 mL (interquartile range = 1500–2500 mL), and four women required no allogeneic blood transfusions. Accidental bladder injury occurred in six women (40%), all of which were successfully repaired intraoperatively without long-term urological sequelae at ≥1 year of follow-up [47]. No maternal deaths occurred. The data demonstrated that focal resection remains highly feasible even in emergency situations and in the presence of advanced, high-grade invasion up to FIGO stage 3b. Histopathological confirmation and intraoperative video documentation validated the reproducibility and accuracy of the technique. The authors concluded that focal excision of PAS allows for high rates of uterine preservation and limited maternal morbidity, comparing favourably with standard caesarean hysterectomy [47].

### 7.4 Endovascular and minimally invasive adjunctive interventions

#### 7.4.1 Endovascular balloon occlusion and pelvic angiography

Prophylactic internal iliac artery balloon occlusion (IIABO) is utilised in numerous specialised centres to reduce intraoperative blood loss, though its reported clinical success rates remain highly variable across the literature [55]. Wright et al. reviewed 20 consecutive women presenting with PAS over a 21-month period, stratifying the cohort by procedure type, total abdominal hysterectomy (TAH) versus conservative myometrial repair (MR), and the utilisation of prophylactic balloon occlusion [55]. The total estimated blood loss was tracked as the primary endpoint.

The analysis revealed that the mean EBL was significantly lower when prophylactic balloon occlusion was implemented. The two-way ANOVA confirmed a significant main effect of balloon use, whereas the primary procedure type exerted no independent statistical effect [55]. However, individual EBL values were widely dispersed: the balloon occlusion cohort demonstrated a median EBL of 1500 mL, while the non-balloon cohort demonstrated a median EBL of 3000 mL, highlighting highly variable therapeutic efficacy at an individual woman level. The authors concluded that while prophylactic balloon occlusion reduces blood loss overall in both TAH and MR for PAS, its real-world efficacy remains variable. Extensive pelvic collateral circulation and individual scar defect morphology likely drive this variability, indicating that future research must refine preoperative risk stratification to guide individualised surgical planning with the aid of advanced imaging techniques [55].

Contrariwise, alternative studies note that prophylactic balloon occlusion (PBO) of the internal iliac arteries has failed to demonstrate consistent, reproducible reductions in blood loss or transfusion requirements [56]. Because static PBO cannot address the extensive, hypervascular collateral neovascularisation characteristic of advanced PAS, several institutions are evaluating the integration of real-time pelvic angiography combined with targeted embolisation to map and occlude these collateral pathways dynamically.

#### 7.4.2 Laparoscopic and surgical devascularisation techniques

Endovascular or surgical adjuncts, including postpartum uterine artery embolisation (UAE), temporary internal iliac balloon occlusion, and surgical ligation of the uterine or hypogastric arteries, can be combined with conservative protocols to control blood loss [48]. UAE is frequently deployed in the acute postpartum setting during expectant management or following an incomplete surgical resection to arrest secondary pelvic haemorrhage. While these devascularisation methods do not replace definitive surgical treatment, they represent valuable tools when immediate haemostasis is required [48].

The gold standard treatment remains caesarean hysterectomy with the placenta left in situ to minimise the risk of catastrophic haemorrhage. Intraoperative medical and surgical measures to reduce blood loss include the early administration of tranexamic acid, aggressive use of uterotonics, and stepwise pelvic devascularisation [48]. Broadly, clinical management of PAS encompasses four distinct frameworks: the extirpative method, caesarean section hysterectomy, conservative expectant management, and one-step conservative surgery, with traditional uterus-preserving approaches historically utilising systemic methotrexate and long-term prophylactic antibiotics [49]. However, conservative management currently yields conflicting data; despite novel pathways for fertility preservation, it poses a significant risk of severe postpartum complications, demonstrating an increased risk of maternal morbidity, mortality, and pelvic infection requiring delayed emergency hysterectomy [50]. While transcervical hysteroscopic resection has been considered for focal lesions, it represents a technically difficult approach due to the presence of large, hypervascular exophytic masses obstructing the lower uterine segment. Open caesarean hysterectomy therefore remains the definitive reference treatment for placenta accreta due to its reproducibility and lower rate of delayed complications relative to conservative options, although it continues to carry a high primary morbidity rate with substantial risks of massive transfusion, accidental cystotomy, ureteral transection, and surgical site infection.

To lower this operative morbidity, several recent case reports have highlighted the utility of laparoscopic management for PAS, demonstrating favourable clinical outcomes with minimal blood loss and low rates of bladder injury [51,52]. The primary keys to laparoscopic success include a structured multidisciplinary approach, the immediate availability of skilled laparoscopic surgeons, advanced surgical equipment, and a tertiary hospital setting [53]. Specific surgical techniques to minimise retroperitoneal complications include comprehensive ureterolysis, direct ligation of the uterine arteries and their contributing collaterals at their origin from the internal iliac arteries, and delaying any physical manipulation of the uterus until complete stepwise devascularisation is achieved. Ureterolysis combined with prophylactic ureteric catheterisation is a vital step to minimise the risk of mechanical or thermal ureteric trauma due to their close proximity to the markedly widened lower uterine segment [54]. Furthermore, delaying the insertion of a uterine manipulator assists in reducing early blood loss, while complete surgical internal iliac artery ligation controls blood flow traversing the placenta via the cervical and vaginal collateral arteries, offering superior haemostatic control compared to isolated uterine artery ligation [54]. When compared directly to open laparotomy, delayed laparoscopic hysterectomy for PAS represents a safe alternative that results in reduced intraoperative blood loss, lower wound infection rates, diminished postoperative pain, earlier patient mobilisation, a shorter hospital stay, and a faster return to daily activities [52].

### 7.5 Delayed interval hysterectomy

Delayed interval hysterectomy refers to performing a planned hysterectomy several weeks after a caesarean birth rather than immediately during the primary birth procedure [57]. This strategy is considered in highly invasive PAS cases where immediate extirpative surgery carries an unacceptably high risk of severe haemorrhage and visceral tissue injury. Clinical evidence indicates that postponing the hysterectomy reduces intraoperative blood loss and decreases total transfusion volumes compared to immediate peripartum hysterectomy. The decision to utilise this approach must be strictly individualised based on the woman’s haemodynamic status and the topographic severity of the invasion, following a thorough multidisciplinary risk-benefit discussion [57].

The underlying clinical rationale for delayed interval hysterectomy stems from the global rise in PAS incidence. Standard management via immediate caesarean hysterectomy is frequently complicated by massive haemorrhage, lower urinary tract injury, and prolonged ICU admissions, with up to 60% of patients requiring the transfusion of four or more units of packed red blood cells, alongside a significant historical mortality rate of up to 7%. Zuckerwise et al. conducted a retrospective cohort study comparing the outcomes of women with an antenatal diagnosis of placenta percreta managed via delayed interval hysterectomy against those undergoing immediate caesarean hysterectomy [57]. The women were treated according to a standardised institutional protocol that included a scheduled caesarean birth at 34–35 weeks of gestation followed by intraoperative multidisciplinary decision-making to determine immediate versus delayed extirpation. In the delayed hysterectomy cohort, the initial caesarean birth utilised a specialised fetal surgery hysterotomy technique to minimise blood loss, leaving the placenta untouched with a plan to perform the interval hysterectomy 4–6 weeks later. The authors collected data regarding demographics, maternal comorbidities, time to interval surgery, estimated blood loss, transfusion requirements, urinary tract injuries, and maternal-fetal mortality [57].

The study identified 49 women with an antepartum diagnosis of placenta percreta, of whom 34 were confirmed to have severe PAS (increta or percreta) at delivery [57]. Delayed interval hysterectomy was performed in 14 women (9 as a scheduled procedure and 5 prior to the scheduled date due to intervening symptoms), while immediate caesarean hysterectomy was completed in 20 women (16 due to an intraoperative assessment of resectability and four due to acute preoperative or intraoperative bleeding). The median estimated blood loss at the time of delayed interval hysterectomy was 750 mL, and the sum total EBL for both the childbirth and the subsequent interval surgery was 1300 mL, which was significantly lower than the median EBL of 3000 mL documented in the immediate hysterectomy cohort (P < 0.01 and P = 0.037, respectively).

Furthermore, the median units of packed red blood cells transfused during delayed interval hysterectomy was 0 units, demonstrating a significant reduction compared to the 4 units required during immediate caesarean hysterectomy [57]. While 45% (9 of 20) of women required the transfusion of ≥ 4 units of red blood cells during an immediate hysterectomy, only 14.2% (2 of 14) required ≥4 units within the delayed interval hysterectomy pathway. One maternal death occurred in each comparative group, representing an individual incidence of 7% in the delayed cohort and 5% in the immediate cohort. The authors concluded that delayed interval hysterectomy represents an effective management strategy for minimising the severity of surgical haemorrhage and reducing the requirement for massive blood transfusions in patients with an antenatal diagnosis of placenta percreta by allowing sufficient time for uterine blood flow to regress and for the placenta to undergo involution away from surrounding pelvic structures [57].

Despite these benefits, delayed interval hysterectomy remains an investigational approach. While data from small operational studies consistently demonstrate lower estimated blood loss (750–850 mL versus 2000–3000 mL), lower total transfusion volumes, and a reduced incidence of large-volume transfusions, the strategy inherently requires two separate major surgeries and potentially multiple hospitalisations [57]. Consequently, it is associated with longer cumulative operative times and an increased total length of hospital stay. Overall rates of major surgical and long-term postoperative complications remain similar to those observed in immediate hysterectomy cohorts, with the most common complications across delayed interval hysterectomy series comprising localised surgical site infections and urinary tract infections [57].

## 8. Complications and outcomes of PAS

### 8.1 Maternal and fetal complications and peripartum hysterectomy associated with PAS

PAS is associated with severe maternal complications, including massive haemorrhage, extensive genital tract and bowel injuries, the necessity for surgical reoperation, and maternal mortality. Fetal and neonatal outcomes are also adversely affected, demonstrating increased risks of prematurity, low birth weight, acute hypoxia, and perinatal mortality. When a patient with PAS delivers, attempts to manually separate the placenta usually trigger massive, torrential bleeding. In low- and middle-income country settings where advanced conservative management therapies might not be readily available, an emergency peripartum hysterectomy is frequently required as a definitive, life-saving intervention to arrest the haemorrhage. Historically, the leading causes of peripartum hysterectomy in low and middle-income countries were uterine rupture, often arising from obstructed labour, and uterine atony. However, with the global and regional rise in caesarean birth rates, PAS has rapidly emerged as a prominent driver of this major surgical intervention [58].

In Nigeria, obstetricians face unique challenges in managing PAS [5, 14]. Factors such as limited access to routine, high-resolution prenatal ultrasound screening mean that many cases are diagnosed late, often dynamically on the operating table during an unexpected emergency delivery [5]. This statistic underlines a critical epidemiological shift, as nearly one in every four peripartum hysterectomies in the studied Nigerian cohort was driven by abnormal placental invasion [5, 14]. This highlights the urgent need for a high index of clinical suspicion through routine screening for risk factors, particularly in women with a history of prior caesarean sections or placenta previa. There is a concurrent need for enhanced utilisation of prenatal Doppler ultrasound imaging to identify abnormal uterine-placental vascularity before labour begins, alongside the establishment of multidisciplinary care teams comprising experienced obstetric surgeons, anaesthesiologists, and blood transfusion services to plan controlled, elective surgeries, which significantly reduces the maternal morbidity and mortality associated with emergency interventions [58].

### 8.2 Impact of placenta previa with PAS on fetal growth

Jauniaux et al. evaluated fetal growth in pregnancies complicated by placenta previa with or without PAS disorder, comparing these outcomes to pregnancies presenting with an isolated low-lying placenta [59]. The authors conducted a multicentre retrospective cohort study of singleton pregnancies complicated by placenta previa with or without PAS disorder, for which maternal characteristics, ultrasound-estimated fetal weight, and birth weight were available. Four maternal–fetal medicine units participated in the collection of data regarding diagnosis, treatment, and outcome. The control group comprised singleton pregnancies with a low-lying placenta situated 0.5–2 cm from the internal cervical os. The diagnosis of PAS and the corresponding depth of invasion were confirmed at birth using both a predefined clinical grading score and histopathological examination. For the comparison of pregnancy characteristics and fetal growth parameters, the study groups were matched for smoking status, ethnic origin, fetal sex, and gestational age at delivery [59].

The study included 82 women with placenta previa with PAS disorder, who were subdivided into adherent (n = 35) and invasive (n = 47) PAS subgroups, alongside 146 women with placenta previa without PAS disorder and 64 controls with a low-lying placenta [59]. The results demonstrated that there was no significant difference in the incidence of small-for-gestational-age (SGA, defined as a birth weight ≤10th percentile) and large-for-gestational-age (LGA, defined as a birth weight ≥ 90th percentile) neonates between the study groups. The median gestational age at diagnosis was significantly lower in pregnancies with placenta previa without PAS disorder than in the low-lying placenta group, but no significant difference was found between pregnancies complicated by placenta previa with PAS disorder and those without for any of the other analysed variables. The median estimated fetal weight percentile was significantly lower in the adherent subgroup compared with the invasive previa–PAS subgroup. However, the actual birth weight percentile at delivery did not differ significantly between these subgroups. The authors concluded that no difference was seen in fetal growth in pregnancies complicated by placenta previa with PAS disorder compared with those without and compared with those with a low-lying placenta. There was also no increased incidence of either SGA or LGA neonates in pregnancies with placenta previa and PAS disorder compared with those with placenta previa with spontaneous separation of the placenta at birth, indicating that adverse neonatal outcome in pregnancies complicated by placenta previa and PAS disorder is linked to preterm birth and not to impaired fetal growth [59].

### 8.3 Neonatal outcomes in pregnancies complicated by PAS

Toussia-Cohen et al. determined the specific neonatal outcomes in pregnancies complicated with PAS compared with pregnancies not complicated by PAS [60]. The authors conducted a retrospective cohort study at a single tertiary centre between March 2011 and January 2022, comparing women with PAS who underwent caesarean delivery to a matched control group of women without PAS who also underwent caesarean birth. The adverse neonatal outcomes assessed included an umbilical artery pH < 7.0, an umbilical artery base excess -12, an Apgar score < 7 at 5 minutes, neonatal intensive care unit (NICU) admission, the need for mechanical ventilation, hypoxic-ischemic encephalopathy, neonatal seizures, and neonatal death. The authors also evaluated a composite adverse neonatal outcome, defined as the occurrence of at least one of these individual adverse neonatal events. Multivariable regression analysis was used to determine which adverse neonatal outcomes were independently associated with the presence of PAS [60].

The study included 265 women with PAS in the study group, who were matched to 1,382 controls. In the PAS group compared with controls, the rate of composite adverse neonatal outcomes was significantly higher. In the final multivariable logistic regression analysis, an Apgar score < 7 at 5 minutes, NICU admission, and the overall composite adverse neonatal outcome were found to be independently associated with the presence of maternal PAS. The authors concluded that neonates born from PAS pregnancies experience significantly higher rates of adverse outcomes, and that an Apgar score < 7 at 5 minutes, NICU admission, and composite adverse neonatal outcomes are independently associated with maternal PAS [60].

## 9. Prevention of PAS

Prevention of Placenta Accreta Spectrum (PAS) can be categorised into primordial, primary, secondary, and tertiary levels [61]. Primordial prevention focuses on reducing the overall pool of at-risk individuals by preventing the initial development of risk factors. This strategy is primarily achieved by reducing unnecessary primary caesarean births through strict adherence to evidence-based obstetric indications, implementing audited labour management protocols, and actively promoting vaginal birth after caesarean section in eligible women.

Primary prevention involves minimizing uterine trauma during necessary surgical procedures to reduce the likelihood of endometrial–myometrial interface defects. This level includes adopting meticulous uterine closure techniques during gynaecological and obstetric surgeries, avoiding aggressive uterine curettage by utilizing medical management for miscarriages where appropriate, and exercising careful clinical decision-making regarding the scheduling of repeat elective caesarean deliveries [61].

Secondary prevention emphasises the early identification of high-risk women during the antenatal period. This requires establishing screening registries for patients with predisposing factors, such as a coexisting history of prior uterine surgery and placenta previa, and utilising high-resolution transvaginal and color Doppler ultrasound to ensure timely referral to tertiary care centres before complications arise.

Tertiary prevention focuses on reducing severe maternal and neonatal complications in women with an established diagnosis of PAS. This strategy relies on minimising blood loss and visceral injury through scheduled, planned births at 34 to 36+6 weeks of gestation, deploying a well-coordinated multidisciplinary care team, and utilising targeted surgical strategies such as planned caesarean hysterectomy, one-step conservative surgery, or delayed interval hysterectomy [61].

## 10. Advances and special clinical contexts in PAS

### 10.1 Contemporary innovations and research horizons in PAS

Recent translational paradigms in PAS feature a highly refined understanding of uterine scar biology and the secondary molecular mechanisms that govern pathological trophoblastic invasion. Diagnostic capabilities have advanced via first-trimester screening protocols, standardised sonographic criteria, and the selective, structured application of magnetic resonance imaging (MRI). The institutional implementation of dedicated, multidisciplinary PAS care teams and standardised clinical guidelines has driven significant improvements in global maternal and neonatal outcomes [62].

Surgical innovations, including modified retroperitoneal hysterectomy techniques and damage control strategies, have further enhanced intraoperative patient safety. Concurrently, interventional radiology applications, such as prophylactic balloon occlusion and pelvic arterial embolization, offer versatile, minimally invasive options for real-time haemorrhage control. Future investigative directions are targeted at novel biomarker development, machine learning and artificial intelligence screening models, and the clinical refinement of fertility-preserving surgical frameworks [62].

### 10.2 Post-myomectomy PAS prevalence and risk stratification

Rojhani et al. conducted a comprehensive systematic review to estimate the true pool prevalence of PAS following prior myomectomy and to evaluate whether specific myomectomy surgical approaches influence subsequent risks of abnormal placentation [63]. The investigators analysed 76 distinct studies comprising 11,065 subsequent pregnancies across 24 countries. The pooled global prevalence of PAS following a history of myomectomy was 1% (95% CI = 1–2%). Prespecified subgroup analyses demonstrated that the absolute prevalence of PAS was highest among women who had previously undergone an open laparotomical myomectomy, presenting a rate of 2% (95% CI = 1–4%; 111/4,474), compared to a rate of 1% following a laparoscopic approach (95% CI = 0–3%; 36/1,172), a rate of 1% following a robotic-assisted approach (95% CI = 0–4%; 4/262), and a rate of less than 1% following hysteroscopic myomectomy (95% CI = 0–1%; 3/271). However, these observed variations between the specific surgical modalities did not reach statistical significance (P = 0.67).

Secondary obstetric outcomes, encompassing postpartum haemorrhage (2%), concomitant placenta previa (1%), placental abruption (1%), and spontaneous uterine rupture (less than 1%)—demonstrated remarkably similar prevalence patterns across all investigated surgical arms. The authors concluded that the baseline prevalence of PAS following myomectomy remains stable at approximately 1% to 2% and does not differ significantly based on the historical surgical approach. Furthermore, because major secondary obstetric complications showed no significant variations among open, laparoscopic, hysteroscopic, and robotic myomectomies, the choice of the primary surgical approach for uterine fibroids can be safely individualised based on baseline patient characteristics, specific myoma topography, and localised surgical expertise [63].

To further contextualise the impact of non-caesarean uterine procedures, Yang et al. performed a large-scale systematic review and meta-analysis evaluating the association between various historical non-caesarean uterine surgeries and the development of PAS in subsequent pregnancies [64]. The analytical framework integrated prospective cohort, retrospective cohort, case-control, and cross-sectional studies involving pregnant cohorts diagnosed with PAS following at least one documented non-caesarean surgical risk factor. The exposures tracked included prior myomectomy, uterine artery embolisation, dilation and curettage, hysteroscopic adhesiolysis, surgical or medical abortion, endometrial ablation, and operative hysteroscopy.

The investigators included 38 studies comprising 7,353,177 individual participants within the systematic review, yielding an overall baseline PAS prevalence of 0.16%, with 31 studies fulfilling the criteria for meta-analysis. The aggregate data demonstrated that prior non-caesarean uterine surgeries are strongly associated with the development of PAS in a subsequent pregnancy, demonstrating a pooled odds ratio of 2.29 (95% CI = 1.43–3.68). When evaluating distinct surgical exposures, highly specific and statistically significant associations with subsequent PAS were identified, ordered by relative risk as follows [64]: Prior uterine artery embolisation demonstrated a pooled odds ratio of 43.16 (95% CI = 20.50–90.88); prior endometrial ablation demonstrated a pooled odds ratio of 20.26 (95% CI = 17.15–23.93), prior hysteroscopic adhesiolysis demonstrated a pooled odds ratio of 7.72 (95% CI = 4.10–14.53); prior operative hysteroscopy demonstrated a pooled odds ratio of 3.10 (95% CI = 1.86–5.18); prior myomectomy demonstrated a pooled odds ratio of 2.29 (95% CI = 1.77–2.97); prior dilation and curettage demonstrated a pooled odds ratio of 2.28 (95% CI = 1.78–2.93) and prior abortion demonstrated a pooled odds ratio of 1.65 (95% CI = 1.43–1.92). These values verify that prior non-caesarean uterine surgeries significantly increase the odds of developing PAS, with the magnitude of the risk varying profoundly based on the specific type of surgical trauma inflicted upon the endometrium and myometrium [64].

### 10.3 Twin gestations complicated by PAS

Di Girolamo et al. conducted a systematic review investigating the specific risk factors, histopathological correlations, prenatal ultrasound detection rates, and maternal-fetal clinical outcomes of twin pregnancies complicated by PAS disorders [65]. The primary outcomes tracked included classic predisposing variables, the specific depth of placental invasion verified via postpartum pathology, the sensitivity of prenatal sonography in multiple gestations, and the incidence of major maternal morbidities such as allogeneic blood transfusion, peripartum hysterectomy, scheduled versus emergency caesarean birth, and maternal mortality [65].

The systematic review synthesised data from two primary studies comprising 103 twin pregnancies complicated by confirmed PAS. Within this cohort, 41.86% (95% CI = 27.0–57.9%) of the twin pregnancies featured a history of prior caesarean birth, 28.22% (95% CI = 13.4–46.0%) presented with a concomitant placenta previa, and 58.14% (95% CI = 42.1–73.0%) were conceived via assisted reproductive technology (ART). Histopathological profiling revealed that 74.49% (95% CI = 41.6–96.5%) of the cases presented as superficial placenta accreta, whereas 25.51% (95% CI = 3.5–58.4%) exhibited deep tissue invasion classified as placenta increta or percreta. A reliable prenatal ultrasound diagnosis of PAS was successfully accomplished in only 27.91% (95% CI = 15.3–43.7%) of the twin cases, a detection rate that is markedly lower than historical sonographic performance documented in singleton cohorts.

Regarding clinical outcomes, which were consistently reported by one large included study, 31.67% (95% CI = 20.3–45.0%) of the women required allogeneic blood product transfusions, 26.67% (95% CI = 16.1–39.7%) required definitive peripartum hysterectomy, and 44.19% required emergency caesarean birth, while zero cases of maternal mortality were recorded. The authors concluded that high-level empirical evidence regarding the natural clinical course of PAS in twin gestations remains limited, noting that while placenta previa, previous caesarean births, and ART represent the dominant risk factors, the prenatal diagnostic accuracy of ultrasound is significantly compromised in twin pregnancies compared to singletons, though approximately 30% of these women will ultimately require blood transfusions and peripartum hysterectomy [65].

### 10.4 Large-scale perinatal and neonatal outcomes of PAS

Viana Pinto et al. evaluated the exact incidence of fetal anomalies, stillbirth, neonatal morbidity, and perinatal mortality in pregnancies complicated by PAS by accessing the international, prospective, multicentre database maintained by the International Society for Placenta Accreta Spectrum (IS-PAS) [66]. Data collection was derived from 23 specialized global centers of excellence, including all confirmed PAS cases across both singleton and multiple pregnancies managed according to institution-specific protocols. The baseline cohort comprised 315 eligible pregnancies with 12 twin gestations, yielding a total of 329 fetuses and newborns; two individual cases were subsequently excluded from final analyses due to structural data inconsistencies regarding fetal abnormalities. For the final calculation of neonatal morbidity and mortality metrics, all elective terminations of pregnancy were excluded, leaving 311 pregnancies and 323 liveborn newborns for rigorous statistical analysis.

Within this prospective cohort, three neonates (0.93%) were stillborn, and among the 320 liveborn infants delivered, 10 cases (3.13%) of early neonatal death were documented. The overall prevalence of major congenital malformations was 4.64% (15/323 newborns), with structural abnormalities localised most frequently to the cardiovascular system, central nervous system, and gastrointestinal tract. The aggregate prevalence of major neonatal morbidity across the entire PAS cohort was 15.1% (47/311 newborns). Interestingly, zero stillbirths, neonatal deaths, or structural fetal malformations were observed within the twin gestation subgroup. The authors concluded that while certain ultra-rare neonatal outcomes may remain difficult to detect within a cohort of this size, these prospective observational data support generally reassuring neonatal outcomes for women surviving PAS pregnancies, confirming that infant risks are largely mediated by iatrogenic prematurity rather than intrinsic placental-mediated growth restriction [66].

### 10.5 Comparative outcomes: prenatal versus intraoperative diagnosis of PAS

Sugai et al. conducted a systematic review and meta-analysis comparing maternal clinical outcomes and specific baseline characteristics between women whose PAS was successfully diagnosed prenatally against those whose condition was discovered unexpectedly intraoperatively [67]. The primary endpoints evaluated included the rate of emergency caesarean birth, definitive hysterectomy, cumulative intraoperative blood loss volume, the number of transfused blood product units, accidental urological injury, consumptive coagulopathy, the necessity for secondary surgical reoperation, and ICU admission, and maternal mortality. Secondary endpoints evaluated maternal age, gestational age at birth, nulliparity, history of prior caesarean birth, history of non-caesarean uterine procedures, utilisation of assisted reproductive technology, the presence of placenta previa, and histopathological confirmation of deep placenta increta or percreta [67].

The meta-analysis synthesized data across 31 comparative studies. The results demonstrated that a successful prenatal diagnosis of PAS is associated with a significantly lower rate of emergency caesarean birth (OR = 0.37, 95% CI = 0.21–0.67), a lower cumulative blood loss volume (mean difference = −0.65 L, 95% CI = −1.17 to −0.13 L), and a significantly reduced requirement for transfused red blood cell units (mean difference = −1.96 units, 95% CI = −3.25 to −0.68 units) compared with the non-prenatally diagnosed cohort. Conversely, the prenatally diagnosed group demonstrated a significantly higher baseline hysterectomy rate (OR = 1.98, 95% CI = 1.02–3.83), reflecting a clinical predisposition toward planned, proactive extirpative management in known cases.

When evaluating secondary clinical markers, the prenatally diagnosed PAS cohort was strongly and statistically associated with prior caesarean birth (OR = 6.81, 95% CI = 4.12–11.25), concomitant placenta previa (OR = 6.81, 95% CI = 4.12–11.25), and advanced placenta increta or percreta (OR = 3.97, 95% CI = 2.24–7.03), while exhibiting negative associations with nulliparity (OR = 0.14, 95% CI = 0.10–0.20) and assisted reproductive technology (OR = 0.19, 95% CI = 0.06–0.61). No significant differences were detected among the remaining maternal endpoints. The authors concluded that despite the intrinsic biological severity of prenatally mapped cases, the highly positive effect of a timely prenatal diagnosis on reducing rates of emergency birth and postpartum haemorrhages underlines the absolute necessity of rigorous antenatal screening, while confirming that prior uterine surgeries significantly elevate subsequent PAS risks in a surgery-specific manner [67].

### 10.6 Comparative efficacy of endovascular prophylactic balloon occlusion in women with PAS

Chen et al. performed a systematic review and meta-analysis evaluating the comparative clinical effectiveness of two specific endovascular interventions deployed during caesarean birth to mitigate haemorrhage in women with PAS: prophylactic balloon occlusion of the abdominal aorta (PBOAA) and prophylactic balloon occlusion of the internal iliac arteries (PBOIIA) [68]. From a primary screening pool of 429 studies, the investigators included 35 clinical trials evaluating these endovascular occlusion modalities. The PBOIIA framework encompassed 19 studies comprising 935 women who underwent internal iliac balloon placement compared against 851 matched controls. The PBOAA framework encompassed 10 studies comprising 428 women who underwent abdominal aortic balloon placement compared against 324 matched controls. Additionally, seven direct comparative studies were analysed, tracking 267 cases of PBOAA directly against 313 cases of PBOIIA [68].

The meta-analysis revealed that the use of PBOIIA achieved a significant reduction in estimated blood loss volume (mean difference = −342.06 mL, 95% CI = −509.90 to −174.23 mL) and a reduction in packed red blood cell transfusion volume (mean difference = −1.57 units, 95% CI = −2.49 to −0.66 units) compared to its respective non-balloon control group. Similarly, the PBOAA cohort demonstrated highly significant reductions in cumulative EBL volume (mean difference = −926.42 mL) and total PRBC transfusion volume (mean difference = −2.42 units, 95% CI = −4.25 to −0.59 units) relative to its respective control arm. When comparing the two active endovascular strategies directly, PBOAA proved significantly more effective than PBOIIA in reducing absolute intraoperative blood loss volume, demonstrating a mean difference of −406.63 mL; however, no statistically significant difference was identified between PBOAA and PBOIIA regarding the total volume of packed red blood cells transfused.

To manage the high statistical heterogeneity observed across the primary meta-analysis, the authors conducted a structured hierarchical analysis stratified by gestational age and maternal age. This hierarchical approach successfully reduced subgroup heterogeneity, identifying both maternal age and gestational weeks at delivery as key confounding drivers of the observed variance. The authors concluded that both prophylactic endovascular balloon techniques are safe and effective methods for controlling obstetric haemorrhage and reducing transfusion requirements in PAS women. Furthermore, PBOAA should be considered the preferred interventional approach for controlling intraoperative bleeding over PBOIIA in institutions possessing the requisite specialised interventional radiologic infrastructure [68].

### 10.7 Intraoperative diagnosis of PAS at term vaginal birth

While the clinical features of antenatally mapped PAS are well documented, the incidental discovery of unexpected PAS during a term vaginal birth remains an ultra-rare event accompanied by severe maternal morbidity. To characterise this specific phenomenon, Rau et al. conducted a large-scale retrospective cohort study utilising the National Inpatient Sample database from the Healthcare Cost and Utilisation Project [69]. The primary study population comprised 8,694,669 term vaginal births recorded over a four-year period. To isolate true incidental presentations, women with a history of prior uterine scarring, documented placenta previa, or preterm birth were strictly excluded from the analysis, with exposure defined exclusively as the intraoperative or postpartum diagnosis of unexpected PAS. The investigators tracked the baseline incidence rate, maternal clinical and pregnancy characteristics, and the relative risk of acute maternal morbidities. Multivariable binary logistic regression and inverse probability of treatment weighting propensity models were utilised for statistical analysis.

The epidemiological data revealed that an unsuspected, incidental diagnosis of PAS occurs in approximately 1 in every 3,797 term vaginal births [69]. Multivariable logistic regression identified several discrete maternal and fetal variables that are independently associated with an increased likelihood of encountering unexpected PAS during vaginal birth: advanced maternal age;, structural uterine factors, specifically congenital uterine anomalies and uterine myomas, acute pregnancy characteristics, including early-term birth and a history of recurrent pregnancy loss and adverse fetal factors, specifically restricted in utero growth and intrauterine fetal demise. Among these variables, the presence of a congenital uterine anomaly demonstrated the strongest independent correlation with unexpected PAS.

In the propensity score-weighted model, women experiencing an unsuspected vaginal PAS delivery faced severe maternal morbidity compared to non-PAS controls, demonstrating a haemorrhage rate of 65.2% versus 4.1%, an allogeneic blood product transfusion rate of 21.3% versus 0.6%, a emergent postpartum hysterectomy rate of 14.9% versus less than 0.1%, an acute consumptive coagulopathy rate of 2.9% versus 0.1%, and an obstetric shock rate of 2.9% versus less than 0.1%. Furthermore, these women required manual removal of the placenta in 25.1% of cases compared to just 0.6% in the non-PAS cohort. The authors concluded that while the incidental discovery of PAS during a term vaginal birth is rare, it is tied to catastrophic maternal morbidity, suggesting that the clear biological association between native uterine anomalies and abnormal placental attachment warrants closer antenatal evaluation [69].

### 10.8 Maternal serum thrombospondin-4 levels in pregnancies complicated by PAS

Çanga et al. conducted a prospective case–control study to evaluate maternal serum thrombospondin-4 (TSP-4) levels in pregnancies complicated by PAS and to assess their correlation with maternal and neonatal outcomes [70]. Performed at Ankara Etlik City Hospital between June 2025 and March 2026, the study enrolled 80 pregnant women, evenly divided between 40 PAS cases and 40 healthy controls. Maternal serum TSP-4 concentrations were quantified using enzyme-linked immunosorbent assay, alongside the collection of comprehensive clinical, obstetric, maternal, and neonatal data. Statistical analyses utilised univariable and multivariable logistic regression alongside receiver operating characteristic (ROC) curves to compare clinical predictive models with and without the integration of TSP-4 [70].

The findings revealed that maternal serum TSP-4 levels were significantly elevated in the PAS cohort compared to the control group (P < 0.001). Within the wider cohort, PAS itself was strongly correlated with adverse outcomes, including prolonged hospitalization, higher transfusion requirements, lower gestational age at birth, reduced birth weight, depressed Apgar scores, and increased rates of neonatal intensive care unit admission, as well as elevated maternal and neonatal composite adverse outcomes. However, within the isolated PAS cohort, TSP-4 concentrations did not significantly correlate with the severity of the PAS grade, composite adverse maternal outcomes, or major neonatal events [70].

Multivariable analysis identified TSP-4 as the sole variable independently associated with PAS (P = 0.001). ROC analysis indicated that TSP-4 possessed moderate discriminative performance, with an area under the curve (AUC) of 0.796 (95% CI: 0.697–0.895). Utilising a diagnostic cutoff threshold of >9.47 ng/mL, the biomarker demonstrated a sensitivity of 72.5% and a specificity of 82.5%, corresponding to positive and negative likelihood ratios of 4.14 and 0.33, respectively. Notably, incorporating TSP-4 into a standard clinical model significantly enhanced its predictive capacity, raising the AUC from 0.660 to 0.816 (P = 0.002 for the difference) [70].

Consequently, the authors concluded that elevated maternal serum TSP-4 serves as a viable, complementary biomarker candidate for antenatal risk stratification when combined with established clinical predictors. Because its diagnostic utility lies primarily in identifying the presence of PAS rather than grading its clinical severity, the authors noted that these preliminary associations require validation through larger, prospective, multicenter cohorts [70].

### 10.9 Re-evaluating conservative uterine retention and serum biomarkers

Regardless of the chosen surgical pathway, achieving optimal clinical outcomes in PAS requires early prenatal detection, meticulous pre-surgical mapping, and the immediate availability of specialised obstetric, anaesthetic, and interventional radiology subspecialties. Because reported success rates for conservative, uterine-preserving approaches vary widely depending on specific outcome parameters, follow-up timelines, and strict patient selection criteria, a direct comparison between individual published series remains challenging. Currently, a planned caesarean hysterectomy with the placenta left entirely in situ is the definitive, standard-of-care management for confirmed high-grade PAS, particularly in women who have completed childbearing. This extirpative strategy is endorsed by major international authorities, including FIGO and IS-PAS, based on the core principle of avoiding manual placental separation, which invariably triggers uncontrollable, life-threatening haemorrhage due to the absolute loss of a physiological decidual cleavage plane [71].

The surgical removal of the uterus should be scheduled electively between 34 and 36 weeks of gestation under stable conditions, prior to the onset of spontaneous labour or acute bleeding episodes. The primary hysterotomy incision must be placed high on the uterine corpus, completely clear of the upper placental border. Following the delivery of the fetus, the uterus is removed en bloc with the untouched placenta left in situ. Major intraoperative risks include massive haemorrhage, bladder transection, and ureteric injury, which frequently require retroperitoneal bladder dissection when placental neovascularisation involves the uterovesical space [44]. Comprehensive postoperative protocols must incorporate intensive care unit (ICU) monitoring, aggressive thromboprophylaxis, and dedicated psychological support due to the high rate of long-term post-surgical somatic and psychological morbidity [44].

Conservative, uterus-preserving frameworks provide an alternative to primary caesarean hysterectomy for a highly select subset of patients who express an earnest desire for future fertility, exhibit well-demarcated focal invasion, or present with extensive pelvic involvement that precludes safe, immediate extirpation [48–50]. These approaches aim to preserve the uterus while minimising acute surgical trauma and maternal morbidity. Contemporary literature identifies four primary uterine-sparing options: expectant management, localised segmental resection, the Triple-P procedure, and focal excision, frequently supported by adjunctive therapies such as systemic methotrexate and uterine artery embolisation [48–50]. Uterine preservation rates approaching 78% to 80% have been documented in specialised centers; however, these figures reflect highly selective women populations, with clinical success traditionally defined as the avoidance of an immediate peripartum hysterectomy rather than long-term reproductive capacity or subsequent obstetric outcomes [48,49].

For women choosing conservative management, the placenta is left untouched in situ after fetal delivery, the umbilical cord is ligated close to its insertion using absorbable suture material, the hysterotomy is closed, and the placenta is left to undergo spontaneous avascular necrosis, autolytic degradation, and eventual resorption over several months [48–50]. This specialised procedure should only be offered to highly compliant patients who can guarantee immediate access to tertiary emergency care and are fully counselled regarding the risks of long-term retention [72]. In unexpected intrapartum settings where an unanticipated PAS is encountered in a resource-limited hospital, this approach can function as a lifesaving temporary first-stage measure, stabilising the woman before a planned, definitive two-step surgery can be coordinated after optimising institutional preparedness [73]. Additional endovascular measures to reduce pelvic perfusion, such as postpartum uterine artery embolisation, can be deployed concurrently [74].

Postoperative management protocols for a placenta left in situ recommend administering intravenous antibiotics for 24 hours, followed by a 4-day course of oral broad-spectrum antibiotics, alongside low-molecular-weight heparin thromboprophylaxis for 10 days. This conservative approach has been shown to reduce immediate operative blood loss by half and iatrogenic urological injuries by one-third compared to immediate caesarean hysterectomy [75]. While successful fertility preservation is documented in 78% of low- to high-grade cases managed in specialist hubs [72], robust high-level evidence supporting this practice in high-grade placenta percreta remains sparse. The largest aggregate review of 60 high-grade percreta cases reported successful uterine retention in 60% of women, but documented a major complication rate of 42% secondary to severe systemic sepsis, delayed catastrophic haemorrhage, or consumptive coagulopathy [76]. Additional risks include extensive tissue necrosis, pelvic abscess formation, choriocarcinoma mimicry, vesicouterine fistulas, deep venous thromboembolism, and acute renal failure. Furthermore, the necessity for prolonged, indefinite clinical follow-up due to uncertainty regarding the optimal timing for secondary intervention remains a primary clinical disadvantage, ensuring that this approach is classified as largely experimental [77].

Currently, there is no high-level consensus regarding how to optimise the postpartum follow-up period for women with placenta left in situ to reduce the risks of sudden septicaemia or delayed haemorrhage. Because secondary blood loss is frequently linked to local neoangiogenesis and placental reperfusion, identifying a clear physiological window for removing the remaining placental mass prior to the onset of extensive neovascularisation is critical. Contemporary literature provides limited evidence for predictive biomarkers capable of tracking real-time neoangiogenesis within the retained placental unit [78]. However, recent data suggest a potential clinical role for tracking longitudinal serum trends of placental growth factor (PlGF), progesterone, beta-human chorionic gonadotropin (beta-hCG), and pregnancy-associated plasma protein A (PAPP-A) to map this therapeutic window [79].

Al Naimi et al. investigated the specific association between these maternal serum markers and the likelihood of achieving successful conservative management in women with high-grade PAS [80]. The authors conducted a retrospective case-control study including women with confirmed high-grade PAS managed between 2011 and 2025 via a standardised conservative protocol designated as the Frankfurt PAS Procedure. In this two-step framework, the placenta was left entirely in situ during the initial caesarean section, followed immediately by prophylactic uterine artery embolisation, with a planned secondary surgery scheduled later to remove the regressed placental mass. Regular postpartum laboratory monitoring tracked sequential serum levels of beta-hCG, PlGF, progesterone, and PAPP-A. Linear mixed-effects models incorporating a random intercept were utilised to evaluate the statistical association between longitudinal marker kinetics and successful, long-term uterine preservation [80].

The study analysed 24 women, 7 of whom achieved successful uterine preservation, while 17 experienced conservative failure and required delayed secondary hysterectomy [80]. The data revealed that the women with successful uterine retention demonstrated significantly lower baseline serum concentrations of beta-hCG, PlGF, and progesterone, exhibiting median values of 1,469 mIU/mL, 117 pg/mL, and 24.5 ng/mL, respectively, compared to the 17 women who failed conservative management, who exhibited significantly higher median values of 9,041 mIU/mL, 227.2 pg/mL, and 60 ng/mL, respectively. No statistically significant differences were detected in PAPP-A expressions between the two groups [80].

The mixed-effects models demonstrated a highly significant, steady physiological decline for beta-hCG, PAPP-A, and progesterone over postpartum follow-up time, whereas serum PlGF levels remained stable without a significant temporal decline. The absolute rate of the serum beta-hCG clearance drop was significantly sharper in patients who achieved successful uterine retention, demonstrating a clearance coefficient drop of 311 mIU/mL per day during the first two weeks of follow-up. The authors concluded that serum beta-hCG, PlGF, and progesterone levels decrease successively following conservative management, and that a strong negative association exists between successful uterine retention and elevated serum beta-hCG kinetics. Based on these dynamic clearance curves, the authors determined that the optimal clinical window for performing a safe, secondary two-step placental removal surgery is precisely 2 to 3 weeks postpartum, a timeframe that minimizes the competing risks of active neoangiogenesis and systemic sepsis [80].

## 11. Future fertility, resource allocations, and molecular horizons in PAS

### 11.1 Fertility and obstetric outcomes following conservative management of PAS

Traditional paradigms presume that definitive peripartum hysterectomy represents the standard approach to minimise the primary maternal morbidity and mortality associated with PAS [81]. Over the past decade, uterine-sparing strategies have been integrated into specialised protocols to preserve long-term fertility and potentially reduce acute surgical trauma. However, while a substantial proportion of women express a clear desire for subsequent conception, high-resolution data regarding real-world reproductive and obstetric outcomes following conservative interventions remain sparse [81].

Javinani et al. conducted a systematic review and meta-analysis to evaluate the long-term obstetric course and maternal safety profiles in women who underwent uterus-preserving management for a confirmed PAS pregnancy [81]. The investigators excluded isolated case reports and aggregated data from all cohort or cross-sectional series tracking the first subsequent pregnancy following any type of conservative treatment. Quantitative synthesis integrated five eligible studies comprising 1,458 participants. The documented conservative techniques included leaving the placenta in situ and localised resection surgery, while the specific uterine-sparing method was not explicitly characterised in three studies [81].

The meta-analysis revealed that the pooled recurrence rate of PAS in a subsequent pregnancy was 11.8% (95% CI = 1.1–60.3%), and 1.9% (95% CI = 0.0–34.1%) of the participants ultimately required a delayed secondary caesarean hysterectomy. Furthermore, PPH complicated 10.3% (95% CI = 0.3–81.4%) of these subsequent gestations, and a composite adverse maternal outcome was identified in 22.7% of the cohort (95% CI = 0.0–99.4%). The authors concluded that while a favourable, liveborn pregnancy outcome is achievable following successful conservative management of an index PAS pregnancy, approximately one out of every four subsequent gestations will experience substantial adverse maternal outcomes. Given this high baseline incidence of recurrence and severe peripartum morbidity, thorough counselling of the women and intensive multi-modal provider preparation remain vital when managing subsequent pregnancies in this specific women population [81].

In a more recent evaluation, Vuong et al. analysed the long-term surgical results of a modified one-step conservative uterine surgery (MOSCUS) protocol in two cases of high-grade PAS, alongside a comprehensive review of subsequent pregnancy outcomes documented between 2020 and 2025 [82]. Two women with a history of severe PAS managed via the MOSCUS technique presented at a tertiary referral center in southern Vietnam with subsequent term and near-term singleton gestations. Both pregnancies achieved highly favourable maternal and neonatal outcomes via planned, elective repeat caesarean birth. Neither spontaneous uterine rupture nor recurrent PAS was observed intraoperatively [82].

High-resolution ultrasound mapping demonstrated that the thickness of the lower uterine segment myometrial layer was preserved at 1–1.5 cm, and both postpartum women and their neonates were discharged uneventfully. Reviewing the contemporary literature, the authors noted that while subsequent pregnancy data after conservative surgery remain limited and highly heterogeneous, maternal-fetal outcomes are generally reassuring. The reported recurrence rate of PAS in a subsequent late pregnancy spans a range from 5.5% to 22.7%, with the primary clinical concerns centered around elevated rates of elective repeat caesarean sections and primary postpartum haemorrhage. The investigators concluded that the MOSCUS framework represents a promising, reproducible method for fertility preservation, but emphasised that a well-resourced antenatal care infrastructure backed by a dedicated multidisciplinary team is necessary to safely navigate these high-risk subsequent gestations [82].

### 11.2 Applicability of international guidelines in low- and middle-income countries on PAS

The clinical applicability of international clinical practice guidelines (CPGs) for PAS is frequently compromised when translated into resource-constrained environments. Nieto-Calvache et al. conducted an international, observational, survey-based study to explore how obstetrician-gynaecologists practicing in LMICs navigate and apply standard CPG recommendations within limited healthcare infrastructures [83]. Experienced clinicians with documented expertise in managing PAS across various LMICs were surveyed to evaluate the feasibility of recommendations issued by four major international consensus panels [83].

Out of 158 expert clinicians contacted, responses were obtained from 65 participants (41.1%), representing 27 middle-income countries. The survey data demonstrated that the real-world management of women with PAS in middle-income settings deviates substantially from the paradigms recommended by international guidelines. The participants identified that their clinical practice was restricted by insufficient hospital infrastructure, low baseline financial resources within local health systems, and an absolute deficit of trained subspecialty multidisciplinary teams (MDTs), rendering adherence to standard CPG bundles impossible.

Furthermore, two-thirds of the surveyed experts reported a total absence of accredited centers of excellence for abnormal placental invasion within their respective nations. In over half of the regional referral hospitals explicitly designated as specialised units for PAS care, functional MDTs did not exist. Consequently, one-third of all patients presenting with an unexpected intraoperative diagnosis of PAS were managed exclusively by the primary surgical team initially performing the delivery, without any additional senior or multi-specialty assistance. The authors of Nieto-Calvache study concluded that the care of PAS women in middle-income countries frequently and significantly deviates from established international recommendations due to structural limitations in local resources and physical infrastructure. These findings highlight an urgent global need to design practical, resource-stratified guidelines and targeted surgical training programmes tailored specifically for low-resource environments [83].

### 11.3 Prophylactic multivessel embolisation and endovascular advanced controls for PAS

Given the variable efficacy rates and potential complications associated with static internal iliac or abdominal aortic balloon occlusion, several advanced centers have shifted toward prophylactic pelvic devascularisation via targeted arterial embolisation. This technique involves selective, prophylactic multivessel embolisation initiated immediately following the birth of the newborn, which aims to reduce cumulative intraoperative blood loss while minimising the risk of non-target tissue ischaemic injury that can arise from prolonged balloon inflation [83, 84]. Similar to the historical data available for balloon occlusion, contemporary prophylactic embolisation registries are primarily retrospective; however, the marked reductions in blood loss and transfusion volumes achieved have driven interest in initiating larger prospective clinical trials [83, 84].

Current consensus guidelines from the International Federation of Gynaecology and Obstetrics (FIGO) [84] and the American College of Obstetricians and Gynaecologists in conjunction with the Society for Maternal-Fetal Medicine (ACOG/SMFM) [9] do not recommend the routine deployment of prophylactic endovascular occlusion for all PAS variants. Both authorities recognise that transcatheter embolisation represents a valuable therapeutic tool during active, uncontrolled postpartum haemorrhage, but maintain that high-level empirical evidence supporting its routine prophylactic use remains limited. Prophylactic balloon occlusion (PBO) of the internal iliac arteries fails to demonstrate a consistent, reproducible reduction in blood loss across unselected cohorts because it does not address the extensive, tortuous collateral neovascularisation characteristic of advanced PAS [84].

To overcome this anatomical hurdle, real-time pelvic angiography combined with selective intraoperative multi-vessel embolisation (SiM-VE) is performed immediately following hysterotomy closure. This sequence effectively targets collateral pelvic supply while avoiding any fetal radiation exposure [84].

Mostajeran et al. conducted a retrospective cohort study spanning 2012 to 2024 to evaluate the clinical association between SiM-VE and perioperative transfusion requirements during planned caesarean hysterectomy for histologically verified PAS [85]. The investigators compared patients undergoing caesarean hysterectomy supported by SiM-VE against a cohort undergoing surgical devascularisation alone. All individuals in the active interventional arm underwent bilateral uterine artery embolisation, with the majority receiving additional, targeted selective embolisation of collateral and neovascular pelvic vessels. The primary study endpoint was the requirement for perioperative packed red blood cell transfusions. Confounding by indication was adjusted using doubly robust inverse probability of treatment weighting, and an Oaxaca-Blinder decomposition analysis was applied to partition and evaluate differences in total operative duration [85].

The study evaluated 268 women, with 234 allocated to the SiM-VE arm and 34 to the surgical devascularisation alone arm [85]. Baseline descriptive statistics revealed that the SiM-VE cohort featured a significantly higher incidence of complete anterior placenta previa (161 [68.8%] versus 14 [41.2%]), a higher prevalence of deeply invasive placenta percreta (109 [46.6%] versus 7 [20.6%]), and lower baseline preoperative haemoglobin concentrations, indicating a substantially higher degree of pre-surgical complexity in the interventional group [85].

In the primary doubly robust multivariate model, the utilisation of SiM-VE was independently associated with a significant reduction in the odds of requiring a perioperative blood transfusion (adjusted odds ratio [aOR] = 0.25, 95% CI = 0.11–0.56). However, the total operative duration was significantly longer in the SiM-VE cohort, with the Oaxaca-Blinder decomposition attributing approximately 30% of this temporal difference directly to the baseline structural complexity of the cases. Additionally, access-site arterial thrombosis requiring emergency surgical intervention occurred in three women (1.3%) within the SiM-VE group. The authors concluded that selective intraoperative multi-vessel embolisation achieves lower adjusted odds of perioperative allogeneic blood transfusion, representing a clear clinical trade-off between reduced blood product exposure and prolonged operative times, accompanied by a 1.3% risk of acute access-site arterial thrombosis [85].

### 11.4 Area-based mapping and PPH prediction in PAS

To optimise pre-surgical risk stratification, recent investigations have focused on refining sonographic methods to quantify the actual topography of abnormal placental attachment. Liu et al. evaluated a novel prenatal ultrasound method designed to estimate the total placenta invasion area, investigating its direct correlation with estimated blood loss (EBL) and adverse maternal outcomes during childbirth [86]. In their retrospective cohort study, the total PAS surface area was measured by calculating the longitudinal length of “tramline sign” obliteration and mapping its geometric distance from the internal cervical os [86]. The physical zone of invasion was segmented into precise trapezoidal sections using 3D Crystal Vue imaging. Linear and multiple logistic regression models were constructed to analyse the predictive validity of this estimated PAS area against primary endpoints, including absolute EBL, standard PPH, severe PPH, and the requirement for allogeneic blood product transfusions after birth [86].

Among the 168 women evaluated, 78 developed PPH and 90 did not [86]. The PPH group demonstrated a greater than two-fold higher mean ultrasound-derived PAS area compared to the non-PPH cohort. Linear regression analysis indicated that every 1 cm^2^ increase in measured PAS area corresponded to a 42.84 mL increase in intraoperative EBL. In multiple logistic regression models, the calculated PAS area functioned as an independent predictor of both standard PPH (adjusted odds ratio [aOR] = 1.08, 95% CI = 1.04–1.13) and severe PPH (aOR = 1.03, 95% CI = 1.02–1.04) [86].

Receiver operating characteristic curve analysis established optimal diagnostic area cutoffs for predicting standard PPH and severe PPH (AUC = 0.83, 95% CI = 0.76–0.89). Utilising these mathematical thresholds, the volumetric PAS area significantly outperformed traditional qualitative ultrasound signs in predicting the severity of peripartum haemorrhage. The authors concluded that 3D Crystal Vue-derived PAS area estimation is highly feasible and correlates directly with intraoperative EBL and secondary PPH risk, providing a reproducible objective metric for pre-surgical planning [86].

### 11.5 Molecular pathogenesis, tissue biomarkers, and interface biology

Emerging data indicate that placental histopathology and high-resolution molecular profiling provide critical insights into the underlying pathogenesis, clinical staging, and prognostic stratification of PAS. Invasive placentation is characterised by the aberrant expression of specific cellular markers that regulate extravillous trophoblast invasion, local angiogenesis, extracellular matrix remodelling, and maternal-fetal immune tolerance. Specifically, dysregulated vascular endothelial growth factor signaling cascades, altered matrix metalloproteinase (MMP-2 and MMP-9) enzymatic activities, and disrupted decidualisation pathways serve as primary molecular drivers of abnormal placental adherence and deep myometrial tissue invasion [87].

Molecular and immunohistochemical evaluations reveal that PAS develops as a direct consequence of a disrupted placental–uterine interface [44]. This pathological microenvironment is characterized by excessive intermediate trophoblastic infiltration accompanied by accelerated, dysregulated local angiogenesis, both of which correlate with the depth of invasion and intraoperative complexity. Furthermore, histopathological data underline that arrested decidual formation and defective remodeling of the maternal–fetal boundary serve as foundational drivers of invasive disease. Consequently, these tissue-based molecular alterations present a potential opportunity for clinical risk stratification, diagnostic refinement, and the proactive prediction of adverse peripartum outcomes [44].

Despite these promising insights, the immediate clinical translation of molecular biomarkers remains restricted. Current research is limited by significant heterogeneity across existing study designs, small single-center sample sizes, and a systemic lack of standardised immunohistochemical assessment protocols. The evaluation of these molecular markers is currently confined to post-birth retrospective histopathology, offering no active utility during routine antenatal screening [44,88]. Realising the diagnostic potential of these molecular targets requires large-scale, prospective multi-center cohorts that integrate tissue-based biomarkers with advanced prenatal imaging modalities and clinical risk profiles. Such multi-modal frameworks are essential to validate prognostic accuracy, facilitate earlier diagnosis, tailor individualised surgical strategies, and safely identify women suitable for uterine-preserving conservative management [44].

### 11.6 Methodological constraints and current literature gaps

While diagnostic imaging and surgical interventions have advanced significantly, substantial deficits persist within the empirical literature for PAS [88]. Diagnostic uncertainty remains a primary operational obstacle; both conventional gray-scale ultrasound and MRI frequently exhibit poor sensitivity when differentiating between focal accretas and deeply invasive percretas [28,64]. This diagnostic ambiguity is compounded by marked variability in radiologist expertise, subjective image interpretation, and atypical or posterior placental locations, culminating in the frequent under- or overestimation of the depth of invasion. Such diagnostic errors directly jeopardise pre-surgical planning and compromise the accuracy of prenatal patient counselling [89].

Furthermore, the long-term sequelae of conservative, non-extirpative management remain poorly characterized due to a lack of standardised post-treatment monitoring protocols and highly fragmented outcome reporting. These clinical gaps are exacerbated by global resource disparities. The deployment of international, guideline-recommended management bundles is frequently impossible in LMICs due to restricted access to subspecialty multidisciplinary teams (MDTs), advanced pelvic imaging modalities, and high-volume massive transfusion protocols [64].

Resolving these deficiencies demands the global implementation of standardised diagnostic frameworks, structured prospective registries tracking conservative interventions, and strategic capacity-building within low-resource environments [90]. Finally, as a narrative review, the conclusions presented herein are inherently constrained by potential selection bias and the lack of a formal quantitative meta-analysis, which may limit the universal generalisability of the synthesised findings [90].

## 12.0 Discussion

### 12.1 Diagnostic integration and risk modeling of PAS

The findings synthesised in this review underline that the management of PAS has transitioned from a reactive intraoperative emergency to a proactive, highly planned multidisciplinary endeavour. The stark rise in global incidence, correlated scale-dependently with escalating caesarean birth rates, highlights a pressing public health concern [6,7]. The diagnostic landscape has been significantly enriched by the characterisation of high-resolution sonographic markers like the rail and tramline signs [36,38]. These signs offer clinicians unprecedented anatomical clarity regarding the depth of villous penetration and lower urinary tract involvement. Furthermore, the integration of computational tools, such as the machine learning models validated by Jochum et al., demonstrates how longitudinal haematological parameters and imaging indices can be synthesized to accurately predict definitive PAS and severe peripartum haemorrhage [33]. This computational risk modeling represents a crucial frontier in standardising care and optimising institutional resource allocation prior to child birth.

### 12.2 Critical appraisal of contemporary management frameworks

A critical appraisal of current management literature reveals a distinct divergence in clinical philosophy regarding optimal surgical timing, the utility of pelvic devascularisation, and the safety of uterine preservation. While consensus guidelines uniformly advocate for definitive caesarean hysterectomy with the placenta left in situ between 34 0/7 and 35 6/7 weeks, implementing this approach globally reveals substantial disparities in resource capability and clinical execution.

The rationale supporting definitive extirpation primarily centers on the complete avoidance of the tissue planes where abnormal neovascularisation compromises surgical margins. However, this “one-size-fits-all” approach has been critiqued for its absolute eradication of reproductive capacity, introducing profound psychosocial morbidity. In response, conservative paradigms have emerged to preserve the uterus; yet, as appraised across multiple cohorts, leaving the placenta in situ shifts the clinical burden from acute intraoperative haemorrhage to delayed, unpredictable postoperative complications, including severe pelvic sepsis and delayed consumptive coagulopathy [34]. Consequently, conservative management requires a highly compliant women population and continuous access to emergency tertiary care, resources that are systematically absent in LMICs.

Furthermore, specialised fertility-sparing frameworks, such as the Triple-P procedure, attempt to balance these extremes by utilising localised myometrial resection combined with temporary internal iliac balloon occlusion. Critiques of the Triple-P procedure focus on its high operational complexity, steep learning curve, and absolute reliance on advanced interventional radiology. In resource-limited healthcare systems, where interventional radiology and massive blood banking reserves are non-existent, the technical pillars of the Triple-P procedure are unfeasible. Thus, while high-income systems deliberate over fine adjustments to fertility-preserving protocols, low-resource settings must rely on rapid, definitive extirpative surgery (such as subtotal peripartum hysterectomy) combined with crude mechanical devascularisation techniques to ensure basic maternal survival. This highlights the necessity for international guidelines to shift away from rigid, resource-intensive mandates and toward adaptable, resource-stratified management frameworks.

## 13.0 Conclusion

PAS has evolved into a critical public health concern requiring accurate prenatal detection and coordinated care. Advances in ultrasound techniques, combined imaging scores, and real-time intraoperative flow assessments facilitate better risk stratification and surgical optimisation. Addressing current limitations through standardised reporting frameworks and expanding access to subspecialty multidisciplinary care, especially in low-resource settings, remains mandatory to reduce severe maternal morbidity and optimise reproductive health preservation.

## Figures and Tables

**Table 1:** Summary of characteristics and main findings of some of the included studies

Author (year)	Study design and population	Key interventions / methodologies evaluated	Main findings and clinical Implications
**Umeh et al. (2024)** [14]	Prospective, three-center cohort study; parturients with prior caesarean sections (≥34 weeks’ gestation).	Evaluation of the impact of short vs. normal Interpregnancy Interval (IPI) on PAS incidence.	Found a PAS incidence of 1.6% in short IPI vs. 0.8% in normal IPI (OR 2.02; 95% CI 0.18–22.13; p = 0.57). Concluded that short IPI does not significantly alter PAS incidence following caesarean birth.
**Imafuku et al. (2024)** [23]	Prospective cohort study; 4,146 pregnant women.	Multivariable analysis of clinical surgical and non-surgical risk factors predisposing to PAS.	PAS occurred in 2.1% of cases. Independent risk factors identified: prior caesarean BIRTH (OR 3.3), dilation and curettage (OR 2.8), hysteroscopic surgery (OR 5.7), uterine artery embolisation (OR 44.1), assisted reproductive technology (OR 4.1), and concurrent placenta previa (OR 13.1).
**di Pasquo et al. (2023)** [25]	Diagnostic accuracy study; pregnant women with placenta previa or low-lying placenta.	Evaluation of a new ultrasound sign: Intracervical Lakes (ICL), defined as hypervascular, tortuous anechoic spaces within the cervix via colour Doppler.	ICL independently predicted major postpartum haemorrhage (OR 3.3), caesarean hysterectomy (OR 7.0), and placenta percreta (OR 2.8). When combined with ≥ 1 classic sonographic sign, the Diagnostic Odds Ratio (DOR) for hysterectomy reached 687.4, indicating its value as a marker for deep villous invasion.
**Hussein et al. (2024)** [26]	Retrospective cohort study; 227 high-risk pregnant women with prior caesarean birth and placenta previa.	Assessment of the diagnostic performance of “intracervical lakes” (via TVS) and the transabdominal “rail sign” in predicting PAS and peripartum hysterectomy (PH).	Retransabdominal “rail sign” increased the odds of PAS at birth. Placental lacunae score of 3+ was the strongest predictor of both PAS (OR 320) and PH (OR 9.00). The presence of ICL strongly anticipated the necessity of PH (OR 12.3).
**Pain et al. (2022)** [28]	Validation cohort study; high-risk pregnant women undergoing prenatal ultrasound and MRI.	Formulation and validation of a combined “Percreta Score” nomogram using ultrasound and magnetic resonance imaging metrics.	Combining ultrasound and MRI parameters using the nomogram significantly improved the positive predictive value (PPV) for differentiating focal placenta accreta/increta from deeply invasive placenta percreta.
**Khwankaew et al. (2024)** [31]	Retrospective, blind observer diagnostic study; 70 singleton pregnancies with placenta previa and suspected PAS.	Accuracy assessment of combined transabdominal ultrasound (TAS) and transvaginal ultrasound (TVS) across operators with varying experience levels.	TAS with adjunctive TVS provided excellent interobserver agreement (kappa = 0.92, p < 0.01). Diagnostic accuracy increased from 81.40% to 94.30% for the lower-experienced operator, and from 94.30% to 97.10% for the highly experienced operator.
**Mitoma et al. (2025)** [32]	Single-center historical cohort study; 52 women with placenta previa.	Intraoperative TVUS evaluation tracking blood flow persistence time in cervical hypervascularity from fetal delivery to signal disappearance.	Mean flow persistence time was significantly longer in PAS cases than non-PAS cases. A threshold of < 7 min ruled out PAS (100% sensitivity), whereas ≥ 14 min yielded a 100% positive predictive value for PAS and 96% specificity for predicting hysterectomy.
**Jochum et al. (2025)** [33]	Case-control diagnostic modeling study; 186 PAS cases vs. 217 high-risk controls.	Evaluation of longitudinal obstetric history, imaging markers, and trimester-specific haematologic index trends using machine learning predictive models.	Confirmed prior caesarean section (OR 1.8) and second- or third-trimester ultrasound markers (OR 28.1 and 27.6, respectively) as dominant predictors. Discovered an inverse association between third-trimester mean platelet volume (MPV) and PAS (OR 0.55), leveraging machine learning to successfully model severe haemorrhage (>1,500 mL).

**Table 2:** Molecular pathways, alterations, and functional consequences driving hyperinvasiveness in PAS

Molecular pathway	Alteration in PAS	Functional consequence
Angiogenesis (blood vessel formation)	Up-regulated vascular endothelial growth factor (VEGF) activity	Triggers extensive, chaotic local hypervascularity and neovascularization. This drives the massive, torrential haemorrhage seen clinically during childbirth.
Cellular adhesion	Altered expression of adhesion molecules, such as E-cadherin	Down-regulation or disruption of these cellular “glues” reduces tissue resistance, permitting the trophoblasts to physically migrate deeper into the muscle wall.
Tissue degradation	Elevated matrix metalloproteinases (MMPs)	Accelerates the breakdown of the extracellular matrix in the myometrium, clearing a physical path for deeper placental penetration.

**Table 3:** Comparative features and areas of difference between the rail and tramline signs

Placenta Accreta Spectrum sign	Diagnostic descriptor	Ultrasound technique	Ultrasound findings	Description of abnormal findings
**Rail Sign** [36]	Placenta increta / percreta severity mapping.	2D color Doppler imaging.	Subplacental and uterovesical hypervascularity accompanied by prominent bridging vessels.	Two distinct parallel lines of dense colour Doppler signals, with connecting bridging vessels appearing as railway sleepers linking the two rails.
**Tramline Sign** [38]	Anatomical depth of invasion: myometrium, uterine serosa, or bladder mucosa.	3D Crystal Vue/Realistic Vue or 3D Silhouette mode.	Structural bisection of the myometrium and bladder perpendicularly in the sagittal plane showing two distinct parallel anatomical boundaries.	

## Abbreviations

ACOG: American College of Obstetricians and Gynaecologists
ART: Assisted reproductive technology
beta-hCG: beta-human chorionic gonadotropin
CPGs: Clinical practice guidelines
EBL: Estimated blood loss
FIGO: International Federation of Gynaecology and Obstetrics
IIABO: Internal iliac artery balloon occlusion
ICL: Intracervical Lakes
IPI: Interpregnancy Interval
IS-PAS: International Society for Placenta Accreta Spectrum
LGA: Large-for-gestational-age
LMICs: Low and Middle-income countries
MDTs: Multidisciplinary teams
ML: Machine learning
MOSCUS: Modified one-step conservative uterine surgery
MR: Myometrial repair
MRI: Magnetic resonance imaging
MPV: Mean platelet volume
NICU: Neonatal intensive care unit
PAPP-A: Pregnancy-associated plasma protein A
PAS: Placenta accreta spectrum
PBO: Prophylactic balloon occlusion
PBOAA: Prophylactic balloon occlusion of the abdominal aorta
PlGF: Placental growth factor
PH: Peripartum hysterectomy
PPH: Postpartum haemorrhage
PROM: Premature Rupture of fetal Membranes
PPROM: Preterm PROM
ROC: Receiver operating characteristic
SANRA: Scale for the Assessment of Narrative Review Articles
SGA: Small-for-gestational-age
SiM-VE: Selective intraoperative multi-vessel embolisation
SMFM: Society for Maternal-Fetal Medicine
TAH: Total abdominal hysterectomy
TAS: Transabdominal ultrasound
TSP-4: Thrombospondin-4
TVS: Tansvaginal ultrasound
UAE: Uterine artery embolisation
VEGF: Vascular endothelial growth factor
